# Landscape genomics reveals genetic signals of environmental adaptation of African wild eggplants

**DOI:** 10.1002/ece3.11662

**Published:** 2024-07-09

**Authors:** Emmanuel O. Omondi, Chen‐Yu Lin, Shu‐Mei Huang, Cheng‐An Liao, Ya‐Ping Lin, Ricardo Oliva, Maarten van Zonneveld

**Affiliations:** ^1^ Genetic Resources and Seed Unit World Vegetable Center Tainan Taiwan; ^2^ Biotechnology, World Vegetable Center Tainan Taiwan; ^3^ Department of Horticulture National Taiwan University Taipei Taiwan; ^4^ Plant Pathology World Vegetable Center Tainan Taiwan

**Keywords:** climate‐resilient, crop wild relatives, environmental stress, genotype–environment association, marker‐assisted improvement

## Abstract

Crop wild relatives (CWR) provide a valuable resource for improving crops. They possess desirable traits that confer resilience to various environmental stresses. To fully utilize crop wild relatives in breeding and conservation programs, it is important to understand the genetic basis of their adaptation. Landscape genomics associates environments with genomic variation and allows for examining the genetic basis of adaptation. Our study examined the differences in allele frequency of 15,416 single nucleotide polymorphisms (SNPs) generated through genotyping by sequencing approach among 153 accessions of 15 wild eggplant relatives and two cultivated species from Africa, the principal hotspot of these wild relatives. We also explored the correlation between these variations and the bioclimatic and soil conditions at their collection sites, providing a comprehensive understanding of the genetic signals of environmental adaptation in African wild eggplant. Redundancy analysis (RDA) results showed that the environmental variation explained 6% while the geographical distances among the collection sites explained 15% of the genomic variation in the eggplant wild relative populations when controlling for population structure. Our findings indicate that even though environmental factors are not the main driver of selection in eggplant wild relatives, it is influential in shaping the genomic variation over time. The selected environmental variables and candidate SNPs effectively revealed grouping patterns according to the environmental characteristics of sampling sites. Using four genotype–environment association methods, we detected 396 candidate SNPs (2.5% of the initial SNPs) associated with eight environmental factors. Some of these SNPs signal genes involved in pathways that help adapt to environmental stresses such as drought, heat, cold, salinity, pests, and diseases. These candidate SNPs will be useful for marker‐assisted improvement and characterizing the germplasm of this crop for developing climate‐resilient eggplant varieties. The study provides a model for applying landscape genomics to other crops' wild relatives.

## Graphical abstract

Environmental selection plays a key role in the genetic variation within African wild relative populations that are both widespread and diverse for climate and soil conditions.

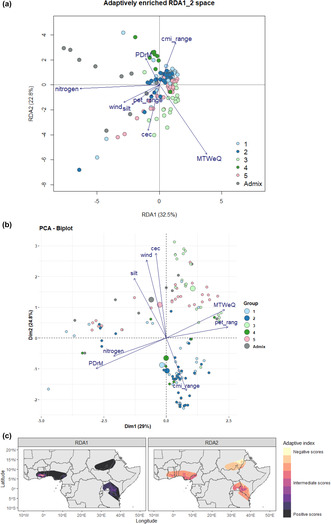

## 1 INTRODUCTION

Crop wild relatives possess traits of interest for breeding climate‐resilient varieties because many are adapted to marginal environments (Kapazoglou et al., 2023). However, it is not often clear what specific adaptive traits they possess and to which abiotic stresses they are adapted (Rellstab et al., 2015), and linkage drag with undesirable traits makes it difficult to detect them (Chitwood‐Brown et al., 2021; Huang et al., 2023). Landscape genomics is an emerging research discipline with a high potential to speed up the detection of valuable traits supporting breeding programs with information about a wide range of associations between specific genome locations and specific environmental factors. Landscape genomics integrates spatial statistics, population genomics, and landscape ecology to rapidly discover various adaptive markers associated with a wide range of environmental factors (Haupt & Schmid, 2022; Manel et al., 2010). It has been successfully applied to detect genes associated with the environmental adaptation of wild plants (Chang et al., 2022; Lasky et al., 2015; Lei et al., 2019; Morente‐López et al., 2018).

Eggplants, including, brinjal eggplant (*Solanum melongena* L.) and African eggplants (*S. aethiopicum* L., *S. anguivi* Lam., and *S. macrocarpon* L.), are important vegetables globally and regionally, belonging to the Solanaceae family. Despite its significance in food production worldwide, eggplant has trailed in the development and use of genomic tools compared to other Solanaceae crops such as potato and tomato (Gramazio et al., 2018). This has changed over recent years due to the development of new genomic resources, including a high‐quality de novo assembled eggplant genome (Barchi et al., 2021; Gramazio et al., 2019). These genomic resources allow us to start screening eggplant genebank accessions for genes associated with environmental adaptation. Crop wild relatives are especially interesting to screen because they possess large untapped genetic diversity and traits of environmental adaptation that disappeared from eggplant varieties during domestication and breeding.

The African wild eggplant populations are particularly interesting because sub‐Saharan Africa is a hotspot of wild relatives of all domesticated eggplants including brinjal eggplant (Aubriot et al., 2018; Syfert et al., 2016). African eggplant wild relatives have shown significant morphological variations and thrive in a myriad of ecological habitats spanning from the equatorial savanna to almost barren desert landscapes (Weese & Bohs, 2010). Most of the African wild relatives belong to either the primary or secondary gene pools of one or more of the domesticated eggplants depending on their phylogenetic relationship and success of crossing with eggplant (Knapp et al., 2013) and are amenable to interspecific hybridization with eggplant (Plazas et al., 2016; Rakha et al., 2020). These *Solanum* species are poorly studied for breeding purposes, and they are underrepresented in seed banks (Syfert et al., 2016). However, it is highly probable that each population has developed specific adaptations to suit their respective local environmental conditions, resulting in many variations.

So far, landscape genomics has not been widely applied to detect genes associated with adaptive traits in crop wild relatives. Traditionally, landscape genomics focuses on single species (Richardson et al., 2016). However, for crop wild relatives, a limited number of records are often available for individual species, and breeders are often interested in screening multiple species in the same crop gene pool for traits of interest (Engels & Thormann, 2020). Therefore, it is common that crop genomic studies cover gene pools with multiple species (Barchi et al., 2021; Lin et al., 2022; Tripodi et al., 2021). This means that by associating genetic signals of multiple crop wild relatives across the environmental gradient of a landscape, we can gain a broad insight into eco‐evolutionary patterns across crop gene pools and identify various options for breeding.

In this study, we apply landscape genomics to screen African eggplant wild relatives for SNPs associated with environmental adaptation. Our objectives were to (i) evaluate the population structure of eggplant wild relatives from diverse environments, taxa, and geographies in West and Eastern Africa; (ii) estimate the contribution of environmental, population structure, and geographic factors in shaping the genomic variation across eggplant wild relatives gene pools; (iii) identify candidate SNPs and their association with the environmental factors. The genotype–environment association was also applied to predict the adaptive landscape; and (iv) investigate the potential role of genes associated with candidate SNPs in enabling local adaptation.

## 2 MATERIALS AND METHODS

### 2.1 Plant material

We genotyped 153 accessions of 17 eggplant species, including 15 wild species and two cultivated species (*S. macrocarpon* and *S. aethiopicum*) collected from wild or feral populations. Royal Botanical Garden, Kew, provided the taxonomic classification, and we further checked in World Flora Online (WFO, 2023). The collections comprised several species representing collections from different West and East African countries collected during the Global Crop Wild Relatives project (http://www.cwrdiversity.org/; Dempewolf et al., 2014, 2017; Müller et al., 2021) and other initiatives. The accession collection points represent different Köppen climate zones (Figure 5; Table S3) (Beck et al., 2018). All accessions are available at the World Vegetable Center (WorldVeg) genebank (https://genebank.worldveg.org/).

### 2.2 Genotyping

According to the manufacturer's instructions, we isolated the genomic DNA from fresh leaves of five seedlings per accession using the FavorPrep Plant Genomic DNA Extraction Mini Kit (FAVORGEN). We then constructed the sequencing library following the approach of Elshire et al. (2011). Genomic DNA was quantified by Qubit and normalized to 100 ng in 96‐well plates. We digested the DNA samples using the restriction enzyme *Ape*KI and ligated them with two adapters for sequencing, followed by the polymerase chain reaction to amplify the target DNA fragments to complete the sequencing library preparation. A service provider did sequencing with the Illumina HiseqX platform in a pair‐end 150 bp run.

For the SNP calling, we followed mainly the manual of Stacks software (Catchen et al., 2013). In short, we filtered the raw reads by quality and demultiplexed using the process radtags program. We then mapped the retained reads to the eggplant reference genome (Eggplant_V4.1.fa) (Barchi et al., 2021) using the Burrows‐Wheeler Aligner (BWA) version 0.7.17 (Li & Durbin, 2009). We sorted and indexed the reads using Samtools version 1.15.1 (Li et al., 2009), after which we performed the variant calling using the gstacks and population programs in Stacks software to obtain a raw VCF of 1,066,587 SNPs. We further filtered the SNPs and the accessions with less than 20% missing data and a Minor Allele Frequency (MAF) >0.05, and LD pruning with a threshold of 0.1 and a window size of 50 bp, giving the final high‐quality SNP dataset comprising 15,146 SNPs used for the analysis.

### 2.3 Environmental data

We selected climate and soil data from three open‐source databases for our models (Table S2). We downloaded the grids for 19 bioclimatic variables, solar radiation, wind speed, and vapor pressure derived from WorlClim 2.1 (Fick & Hijmans, 2017) at a resolution of 2.5 min. The 19 bioclimatic variables were each downloaded as annual data averages between 1970 and 2000. We averaged the monthly solar radiation, wind, and vapor pressure rasters to obtain annual value rasters from this period. We complemented the climate data set with a set of climate‐related variables downloaded from CHELSA (Climatologies at high resolution for the earth's land surface areas), a downscaled climate data set at a resolution of 30 arcsec (~1 km) globally starting from 1980 until 2018 (Brun et al., 2022). The variables included vapor pressure deficit, potential evapotranspiration, climate moisture, growing degree days, growing season length, growing season temperature, and growing season precipitation. Soil variables included nitrogen, soil organic carbon, organic carbon density, organic carbon stock, cation exchange capacity, pH, clay sand, and silt content. We downloaded the soil data from the SoilGrids database released in 2016 (https://soilgrids.org/) through ISRIC—WDC Soils (Hengl et al., 2017) at 250‐meter resolution and at a depth of 15–30 cm, approximately the depth at which the eggplant roots can grow. We aggregated the resolution of the soil dataset to match that of the climate data, ensuring they are consistent in both resolution and extent. We averaged the aggregated soil values using the *resample* and *extent* functions of the raster package in R (Hijmans, 2023).

For each accession, we extracted the data of the environmental variables with the *extract* function of the R raster package (Hijmans, 2023) using the GIS coordinates at sampling points to obtain a full data set of all the climate and soil variables. For the modeling, we selected the environmental variables based on variance inflation factors (VIFs). VIFs under five are considered low correlation (James et al., 2013). We selected a final set of eight climate and soil variables with very low correlation for downstream analysis (Table S1, Figure S5). Following the selection of environmental variables, we tested our hypothesis of isolation by the environment (IBE) versus isolation by distance (IBD) using the mantle test *mantel.rtest* function in the R package ade4 (Dray & Dufour, 2007).

### 2.4 Population structure and differentiation analysis

We used the program STRUCTURE ver.2.3.5 (Pritchard et al., 2000) and *snmf* function in the R package LEA (version 1.4.0) (Frichot et al., 2014) to investigate the population structure. We assigned the 17 species as our populations in the STRUCTURE analysis. These species represented seven eggplant clades (the Melongena, Anguivi, Arundo, Coagulans, Giganteum, Acanthophora, and Aculeastrum clades) (Aubriot et al., 2016; Syfert et al., 2016; http://www.solanaceaesource.org). Considering this phylogenetic structure, we ran sub‐populations varying from *K* = 2 to *K* = 10, each with 10 independent runs and a burn‐in of 10,000 iterations for each run. To determine the optimal *K*, we generated Delta (Δ) *K* for each *K* with a web‐based program, STRUCTURE HARVESTER ver0.6.94 (Earl & von Holdt, 2012). We then aligned and plotted Delta *K*‐s against *K*‐s with the CLUMPAK server (Kopelman et al., 2015). For comparison to the structure analysis, we also estimated the ancestry coefficients using the function *snmf* in the LEA R package. For snmf, we ran sub‐populations from *K* = 1 to *K* = 10 with 10 repetitions for each run. The best *K* was chosen using the cross‐entropy criterion of all the runs. The admixture coefficients (Table S1) for the optimal *K* value for each individual were plotted as pie charts using the *map* function in the maps R package (Becker et al., 2022) onto the map showing the sampling site. We also constructed a dendrogram based on the SNP markers using the Unweighted Pair‐Group Method with Arithmetic Averaging (UPGMA) in TASSEL v5.2.89 (Bradbury et al., 2007) and plotted using TreeViewer (Bianchini & Sánchez‐Baracaldo, 2024).

We analyzed the genetic differentiation by computing *F*_ST_ and AMOVA between genetic groups identified through population structure analysis. We computed *F*_ST_ values and AMOVA using *genet.dist* function in the R package hierfsat (Goudet & Jombart, 2022) and *poppr.amova* function in R package poppr (Kamvar et al., 2015) respectively. We further dissected the genetic variation within the genetic groups. We calculated three measurements of diversity (nucleotide diversity (*π*), Watterson's theta (*θ*), and Tajima's *D*) in TASSEL v5.2.89 (Bradbury et al., 2007) using a step size of 100 bp and window size of 500 bp.

### 2.5 Partitioning of the genomic variation and identification of candidate SNPs

We used four genome scan methods to make a complimentary selection of candidate SNPs among the different methods.

First, we used simple redundancy analysis (RDA) to associate the obtained SNPs with the selected environmental factors without controlling for population structure and geographical distances. RDA is a multivariate method for assessing a linear relationship between two or more factors (Legendre & Legendre, 2012). RDA was performed with the *rda* function of the R package vegan (Oksanen et al., 2022). We carried out 5000 permutations to test the significance of explanatory variables with the R function *anova.cca*.

Second, we used partial RDA, which allows the partitioning of genomic variation into components explained by different factors. To partition the genomic variation into these components, we conducted a full model RDA with the selected environmental factors, spatial autocorrelation, and population structure and then did a partial RDA conditioned on covariates to estimate the proportion of SNP variation explained by the factors that were included in the model. We did the partial RDA with the *rda* function and the number of permutations as the simple RDA explained above. To account for the effect of spatial autocorrelation on SNP variation in the partial RDA analysis, we applied distance‐based Moran's eigenvector maps (dbMEMs) in RDA (Dray et al., 2006; Legendre & Legendre, 2012). This involved first building a neighborhood connection network of 153 collection points. With this network, we constructed a spatial weighting matrix of inverse geographical distances (km^−1^) following the method by Forester et al. (2018). The spatial weighting matrix was then decomposed to generate dbMEMs. Subsequently, we performed forward selection with the *forward*.*sel* function (Dray et al., 2022) to identify dbMEMs that associate significantly with spatial genetic structure. We then applied the selected dbMEMs in RDA to capture comprehensive spatial autocorrelation (Table S5). To account for the effect of population structure on SNP variation in the partial RDA analysis, we used the ancestry coefficients estimated by the STRUCTURE program with the optimal *K* (*K* = 8) as covariates. In RDA, SNP outliers were defined as the SNPs having loadings along the first three RDA axis ±3 SDs from the mean for each axis following Capblancq et al. (2018).

In the preliminary analysis, we noticed that population structure clustering was largely related to eco‐geographical habitats. To separate the connections of population structure to environment and geography, we calculated the proportion of population structure attributed to environmental factors and spatial autocorrelation. We substituted the SNPs with the ancestry coefficient from the STRUCTURE analysis as our new response variable in the RDA models. Our new response variables in the RDA models were the ancestry coefficients from the STRUCTURE analysis with the optimal *K*.

Third, we performed latent factor mixed model (LFFM), a univariate method to associate the obtained SNPs with the selected environmental factors. LFFM allows control for population structure using the R package lffm (Caye et al., 2019). In the LFMM analysis, we controlled the population structure using the optimal *K*, which we initially determined using the STRUCTURE program and *q* values computed. We considered SNPs with a false discovery rate (FDR) <0.05 to be candidate SNPs.

Fourth, we used PCAdapt as an outlier differentiation method. The R package PCAdapt uses a PCA‐based approach to simultaneously infer population structure and identify outlier loci related to this structure. In contrast to the three previous methods, it does not return associations between obtained SNPs and the selected environmental factors (Luu et al., 2016). With this different approach, we expected to capture other candidate SNPs not yet captured by the three gene–environment association methods explained above. We adjusted the *p* values using the Bonferroni method in the *p.adjust* function of the R stats package (R Development Core Team). After that, we applied an FDR ≤0.05 as the significance level for detecting the outlier loci.

### 2.6 Prediction of adaptive landscapes with a selected set of candidate SNP markers

After identifying the candidate SNP markers and their associated environmental factors from all four methods mentioned, it is possible to predict the level of candidate SNP markers in a specific environment. The model shows the geographic patterns in environmental adaptation on a grid map of a so‐called adaptive landscape (Capblancq & Forester, 2021). We carried out a simple RDA to model the adaptive landscape with the set of candidate SNPs—from the four methods explained above—and a set of environmental variables most strongly correlated with the putative adaptive variation. In this case, we used the candidate SNPs as the multivariate response in the simple RDA using the selected environmental variables as the explanatory variables. After that, we calculated an adaptive index for each geographic grid cell of the adaptive landscape based on the genotype‐environment associations following the procedure outlined by Steane et al. (2014). The adaptive index provides an estimate of the adaptive similarity or difference of all the grid cells in the landscape as a function of the environmental predictor values of each grid cell (Capblancq & Forester, 2021). We geographically mapped the indices for RDA axes 1 and 2 using the R package ggplot2 (Wickham, 2016). Visualizing the adaptive landscape enabled us to observe the geographic distribution of the adaptive alleles across the population ranges of the crop wild relatives involved. A higher positive or negative adaptive index score is associated with changes in allele frequency of candidate SNPs across environmental gradients. The modeled landscape was limited to the geographic areas of 153 populations of eggplant wild relatives using the st_*convex_hull* function of the R package sf (Pebesma, 2018). We extended the convex hull at a distance equal to 1 using the *st_buffer* function.

### 2.7 Gene annotation

We identified genes linked to the candidate SNPs using the Sol Genomics Network data file transfer protocol (FTP) database for the eggplant genome consortium version 4.1 (Barchi et al., 2021) (https://www.solgenomics.net/ftp/genomes/Solanum_melongena_V4_Pangenome/Annotation_V4/). The candidate gene search and Gene Ontology (GO) terms were assigned using the *Arabidopsis* information resource (tair) (https://www.arabidopsis.org/) and Uniprot (https://www.uniprot.org/) databases. We characterized the genes and their functions, particularly those associated with abiotic stress and relevant to the environmental adaptation of the eggplant wild relatives.

## 3 RESULTS

### 3.1 Population structure

**FIGURE 1:**
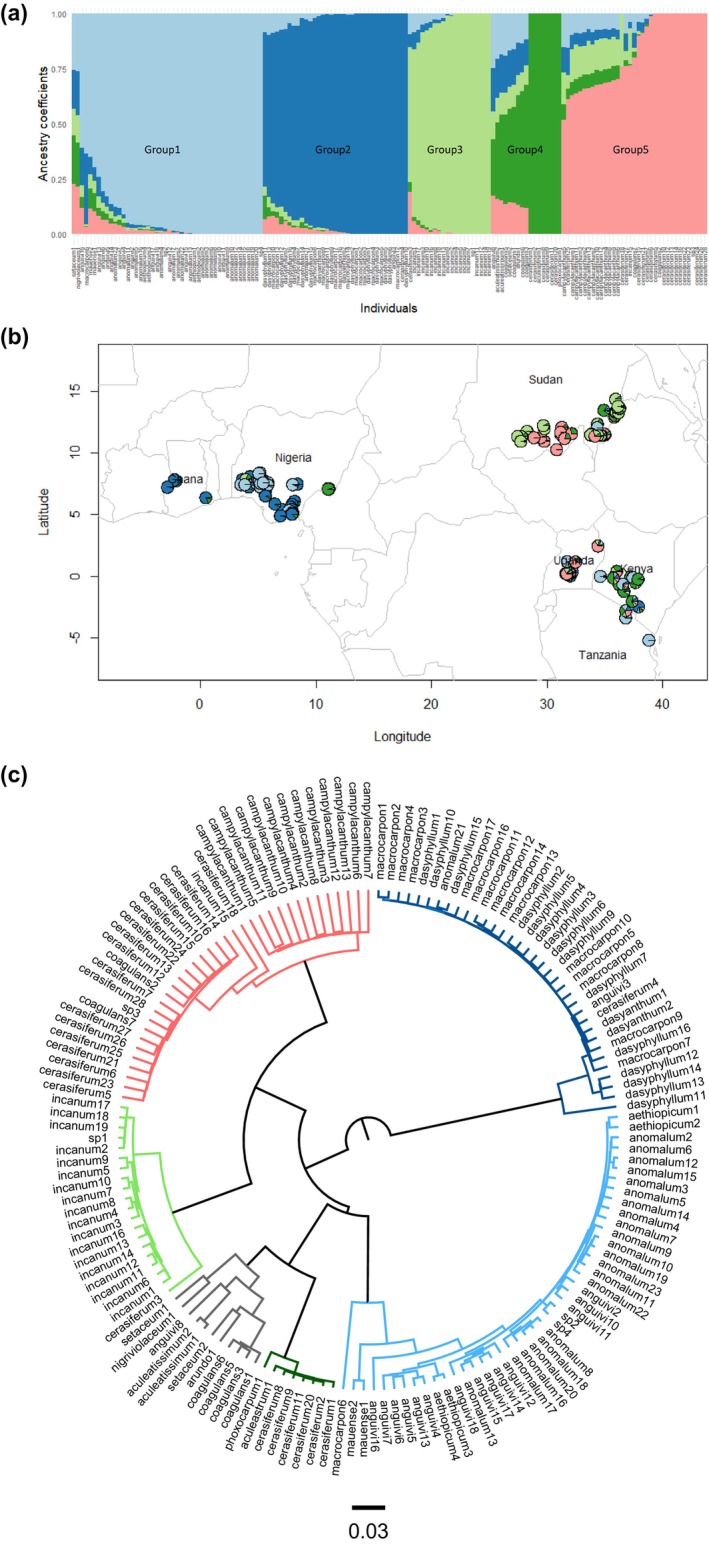
(a) The group formations with sorted *q* values of the ancestry coefficient matrix. The bar plot represents individuals; (b) a map of the sampling areas in Western and Eastern Africa with pie charts showing the admixture proportions from structure analysis (optimal *K* = 5); (c) a dendrogram with colors corresponding to the five genetic groups formed in the bar plots of the population structure analysis. The groups comprise species from different collection populations and gene pools. The main species for every group included Groups 1—*S. anomalum*, *S. incanum*, and *S. aethiopicum*; Group 2—*S. macrocarpon*, *S. dasyphyllum*, *S. anomalum*, and *S. incanum*; Group 3—*S. cerasiferum* and *S. anguivi*; Group 4—*S. anguivi* and *S. anomalum*; and Group 5—*S. campylacanthum*. Groups 1 represents the Coagulans, Acanthophora, and Arundo clades; Groups 2 and 5 represent the Anguivi and Giganteum clades, while Groups 3 and 4 represent the Melongena and Aculeastrum clades. Admixtures were observed mainly in Groups 1 and 3 for accessions of *S. incanum*, *S. cerasiferum*, *S. coagulans*, and *S. nigriviolaceum* species.

The STRUCTURE harvester calculated an optimal number of eight groups (*K* = 8) (Figure S1). Five groups were evident (Figure 1; Figure S3), but optimal *K* = 8 from the STRUCTURE analysis could be due to unclarified groups arising from admixtures. The sNMF analysis also confirmed five groups (Figure S2). Therefore, we considered the ancestry coefficients in the clustering with *K* = 5 optimal and applied them in the genome scan analysis of RDA and LFFM. We also identified several admixed individuals from the hierarchical population structure plot at *K* = 5 for almost all the species (Figure 1a). From *K* = 2 to *K* = 10, the cultivated *S. macrocarpon* and its wild ancestor *S. dasyphyllum and S. dasyanthum* separated from the other 14 species (Figure S3). This group belongs to the Anguivi clade, and the accessions are mainly from the West African eggplant populations.

**FIGURE 2:**
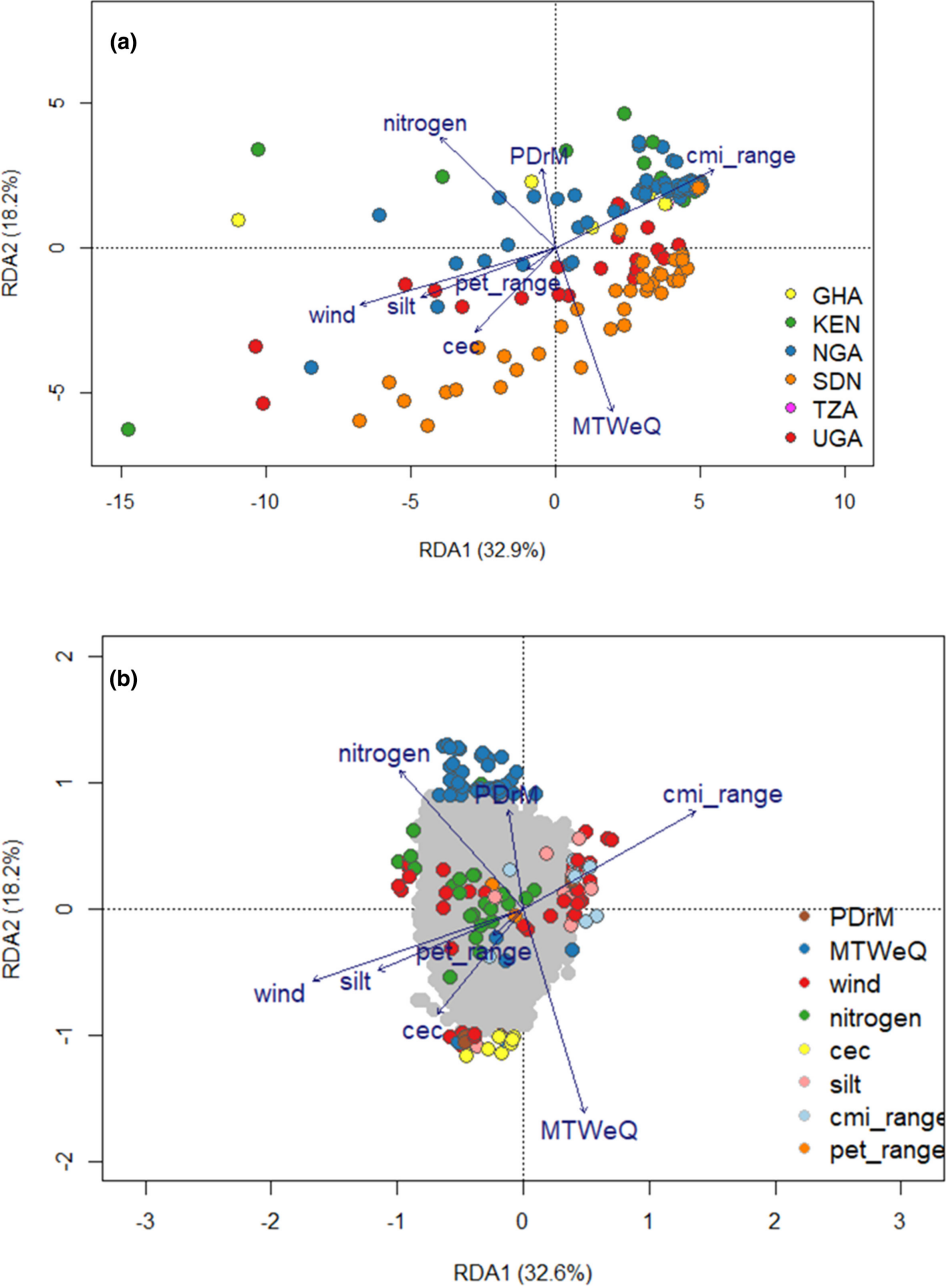
Biplot of simple RDA. Blue vectors indicate the direction and values of the environmental variables. (a) Colors correspond to the individual accession sampling sites by country. TZA: Tanzania; UGA: Uganda; KEN: Kenya; SDN: Sudan; NGA: Nigeria; GHA: Ghana; (b) Outlier SNPs colors correspond to the environmental variable with the strongest association. PDrM: Precipitation of the driest quarter; MTWeQ: Mean temperature of the wettest quarter; wind: wind speed; cmi_range: Annual range of monthly climate moisture index; pet_range: Annual range of potential evapotranspiration; nitrogen: Soil nitrogen content; silt: Soil silt content; cec: Cation exchange capacity.

The dendrogram based on the SNPs displayed five genetic groups (Figure 2c), supporting the clustering in the population structure analysis. While the groups represent seven *Solanum* clades, they largely reflect the geographic regions where the populations were sampled (Figure 1b). Groups 1 and 2 comprise West African accessions and *S. macrocarpon*, *S. dasyphyllum*, and S. *anomalum*. Groups 3, 4, and 5 are mostly East African groups, and the species include *S. incanum*, *S. cerasiferum*, *S. aethiopicum*, *S. setaceum*, *S. campylacanthum*, and *S. aculeatissimum*.

The observed and expected heterozygosity averages were 0.01 and 0.07, respectively. The inbreeding coefficient within the groups ranged from. 0.79 for Group 3 to 0.94 for Group 4 (Table 1). The genetic differentiation results showed a moderate level of genetic differentiation among all the groups. The values ranged from 0.50 (between Group 4 and the admixed groups) to 0.91 (between Group 1 and Group 3) (Figure S4). This indicates significant differentiation between the groups. The AMOVA results showed that the genetic differentiation between the groups and within groups accounted for 80.92% and 19.08 (*p* ≤ .01) of the total variation, respectively, indicating a high genetic diversity between the populations as opposed to within populations (Table S4). Nucleotide diversity (*π*) and Watterrson's theta (*θ*) were highest in Groups 2, 4, and 5, and the admixed group with a *π* range of 0.08–0.10 and *θ* range of 0.09–0.15. The nucleotide diversity was lowest in Groups 1 and 3 at 0.04 and 0.03, respectively. The low nucleotide diversity is also supported by the low expected heterozygosity for these groups. All the groups showed a negative though differing Tajima's *D* values. Group 5 had the highest value (−0.29), while Groups 1 and 4 had the lowest value (−1.80). The negative Tajima's *D* values indicate selective sweeps in the populations of the wild eggplant species.

**TABLE 1:** Population genetic parameters for African eggplant wild relatives groups based on SNPs.

Groups	Main regions in each group	*N*	Private alleles	*H*_O_	*H*_E_	*F*_IS_	*π*	*θ*	Tajima's *D*
1	West Africa	41	12,499	0.01	0.04	0.83	0.04 (0.003)	0.07 (0.005)	−1.78 (0.140)
2	West Africa	38	100,029	0.01	0.08	0.92	0.08 (0.004)	0.13 (0.005)	−1.25 (0.095)
3	East Africa	20	22,873	0.00	0.03	0.79	0.03 (0.006)	0.04 (0.006)	−1.29 (0.351)
4	East Africa	9	17,175	0.00	0.10	0.94	0.10 (0.006)	0.15 (0.010)	−1.80 (0.067)
5	East Africa	34	7168	0.01	0.08	0.85	0.08 (0.010)	0.09 (0.008)	−0.29 (0.223)
Admix	East Africa	11	452	0.00	0.09	0.92	0.10 (0.009)	0.11 (0.009)	−0.54 (0.343)

*Note*: Standard deviations for *π*, *θ*, and Tajima's *D* are provided in the brackets.

Abbreviations: *F*_IS_, inbreeding coefficient; *H*_E_, expected heterozygosity; *H*_O_, observed heterozygosity; *N*, number of individuals; *θ*, Watterson's Theta; *π*, Nucleotide diversity (pi).

### 3.2 Genomic environmental association analysis

Among the eight non‐redundant environmental variables, we identified using VIF with a threshold of five, PDrM (precipitation of the driest quarter), MTWeQ (mean temperature of the wettest quarter), wind (wind speed), cmi_range (annual range of monthly climate moisture index), and pet_range (annual range of potential evapotranspiration) describe the precipitation and temperature patterns, while nitrogen (soil nitrogen content), silt (soil silt content), and cec (cation exchange capacity) described the soil properties of the sampling sites. The results of the Mantel test revealed a significant positive correlation (*r* = .083, *p* < .0004) with the spatial distances of our sampling points (Figure S8), implying that the further the geographic distances, the greater the environmental dissimilarities based on the selected environmental variables.

### 3.3 Redundancy analysis models and the genomic variation partitioning

**FIGURE 3:**
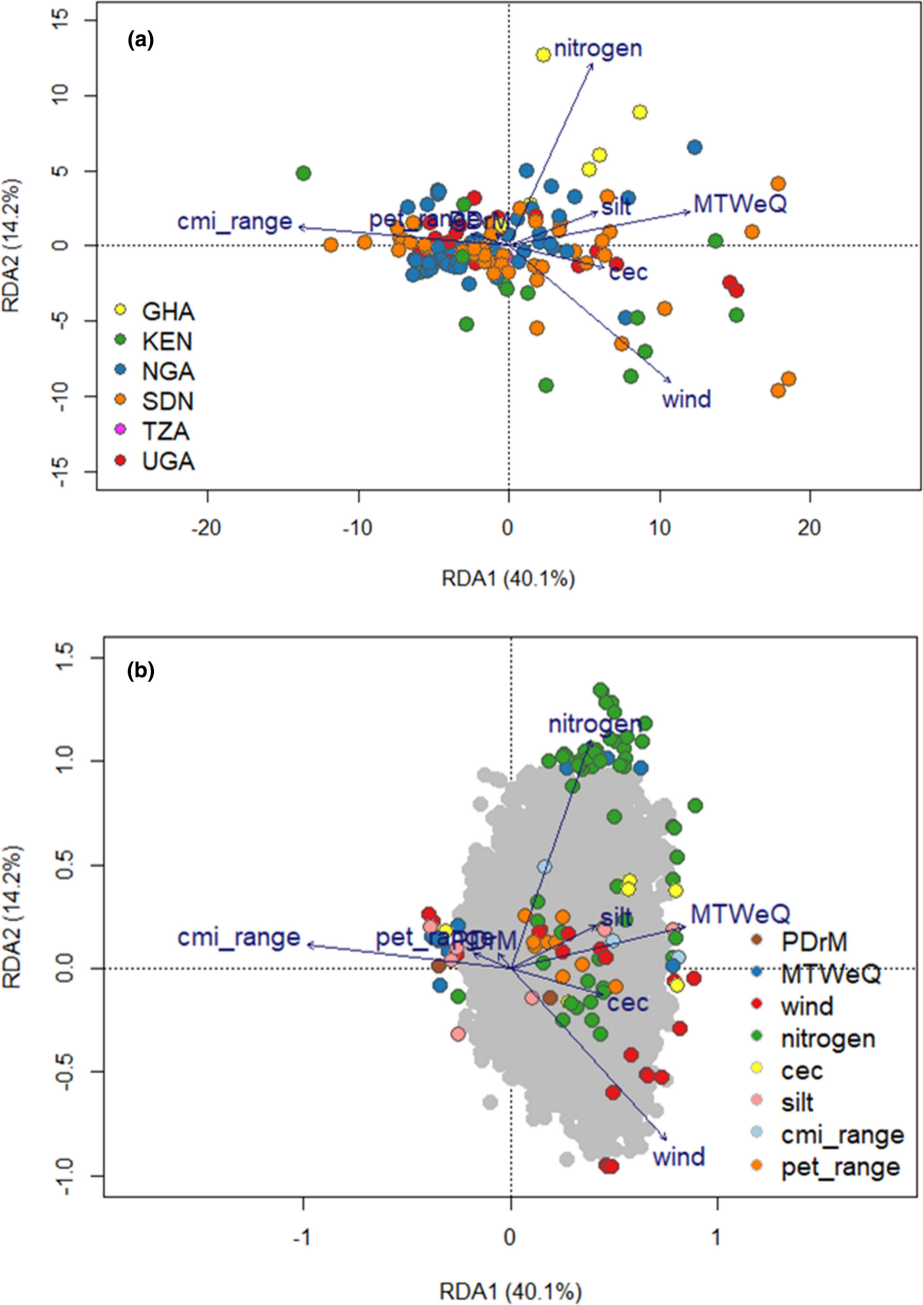
Biplot of partial RDA conditioned on geographical distances and population structure. Blue vectors indicate the direction and values of the environmental variables. (a) The individual accessions are highlighted in color based on the origin. TZA: Tanzania; UGA: Uganda; KEN: Kenya; SDN: Sudan; NGA: Nigeria; GHA: Ghana; (b) Outlier SNPs are highlighted in color based on the environmental variable with the strongest correlation. PDrM: Precipitation of the driest quarter; MTWeQ: Mean temperature of the wettest quarter; wind: wind speed; cmi_range: Annual range of monthly climate moisture index; pet_range: Annual range of potential evapotranspiration; nitrogen: Soil nitrogen content; silt: Soil silt content; cec: Cation exchange capacity.

All our RDA models were significant (*p* ≤ .002 and .000 for sRDA and pRDA, respectively). The simple RDA showed genetic differentiation within countries of origin on the first axis (Figure 2a). The second axis showed genetic differentiation among the populations among the populations with accessions from Uganda, forming a central group between accessions from Nigeria, Ghana, Kenya, and Sudan. These results suggest a significant environmental effect on genetic differentiation. This observation is also consistent with the STRUCTURE analysis results that showed clustering primarily due to environmental characteristics. The highest biplot scores on the first RDA axis were for wind and cmi_range (−0.67, 0.55, respectively: Table S5). The highest scores on the second RDA axis were for the mean temperature of the wettest quarter (−0.75) and soil nitrogen (0.51). The simple RDA identified one hundred and thirty‐nine candidate SNPs on the first three RDA axes, which explained 61.5% of the SNP variation. Thirty‐nine candidate SNPs were detected on the first axis, while 59 and 44 candidate SNPs were detected on the second and third axes, respectively. Most of the SNPs were associated with the mean temperature of the wettest quarter, wind, and soil nitrogen (44, 37, and 27, respectively; Figure 2b; Table S6). Conditioning the RDA on population structure and geographical distance significantly reduced the effects of environmental variables compared to the simple RDA (Figure 3a; Table S6), indicating a high correlation between environmental factors and other factors (geographic distances and population structure). The highest biplot scores on the first RDA axis were for wind and cmi_range (−0.67, 0.55, respectively: Table S5). The highest scores on the second RDA axis were for the mean temperature of the wettest quarter (−0.75) and soil nitrogen (0.51). The simple RDA identified one hundred and thirty‐nine candidate SNPs on the first three RDA axes, which explained 61.5% of the SNP variation. Thirty‐nine candidate SNPs were detected on the first axis, while 59 and 44 candidate SNPs were detected on the second and third axes, respectively. Most of the SNPs were associated with the mean temperature of the wettest quarter, wind, and soil nitrogen (44, 37, and 27, respectively; Figure 2b; Table S6).

The pRDA further decomposed the contribution of environmental, geographic distance, and population structure in explaining the inter‐population genetic variation. All the variables jointly explained 55.0% of the genetic variation (Table 2). The collinear portion of the environment, geographic distances, and population structure accounted for most (28.0%) of the total explained variation. Independently, the environment and population structure accounted for 6.1% and 5.4% of the total genetic variation, respectively, while geographic distances explained the largest fraction of the variation at 15.0%.

**TABLE 2:** The contribution of the environmental variables (climatic and soil), population structure, and geographical distances to the genomic variation and observed genetic structure in the partial RDA models.

	Models	*R* ^2^	Adj *R* ^2^	*p* (>*F*)	Proportion of total variance
SNP variation	Full: Y ~ Env + Struct. + spatial	.282	.148	.000***	0.28
	Y ~ Env + | (Struct. + Spatial)	.061	.025	.500	0.06
	Y ~ Struct. | (Env + Spatial)	.054	.030	.229	0.05
	Y ~ Spatial | (Env + Struct.)	.154	.094	.009**	0.15
	Confounded Env. /Struct. / Spatial				0.01
	Total explained				0.55
	Total unexplained				0.45
Structure	Struct. ~Env. + Spatial	.353	.255	.000***	0.35
	Struct. ~ Env. |(Spatial)	.259	.248	.000***	0.26
	Struct. ~Spatial |(Env)	.078	.036	.000***	0.08
	Confounded Env. / Spatial			—	0.01
	Total explained			—	0.70
	Total unexplained			—	0.30

*Note*: Env = Environmental variables; Spatial = Geographical distances (spatial autocorrelations); Struct. = Population structure. *p ≤ .05, **p ≤ .01, ***p ≤ .001

Since genetic clusters largely corresponded to geographical areas, we partitioned the population structure to determine the proportions explained by environmental factors and geographical distances between the sampling points. Collinear factors of the environment and geographic distances significantly explained 35.3% of the total explainable population structure. The environmental factors alone accounted for 25.9% of the population structure, while geographical distances only accounted for 7.8%. These results suggested that isolation by environment explains the population structure of the wild eggplants. This also aligns with the results of the Mantel test that showed environmental differences with increased spatial distances.

### 3.4 Candidate SNPs

**FIGURE 4:**
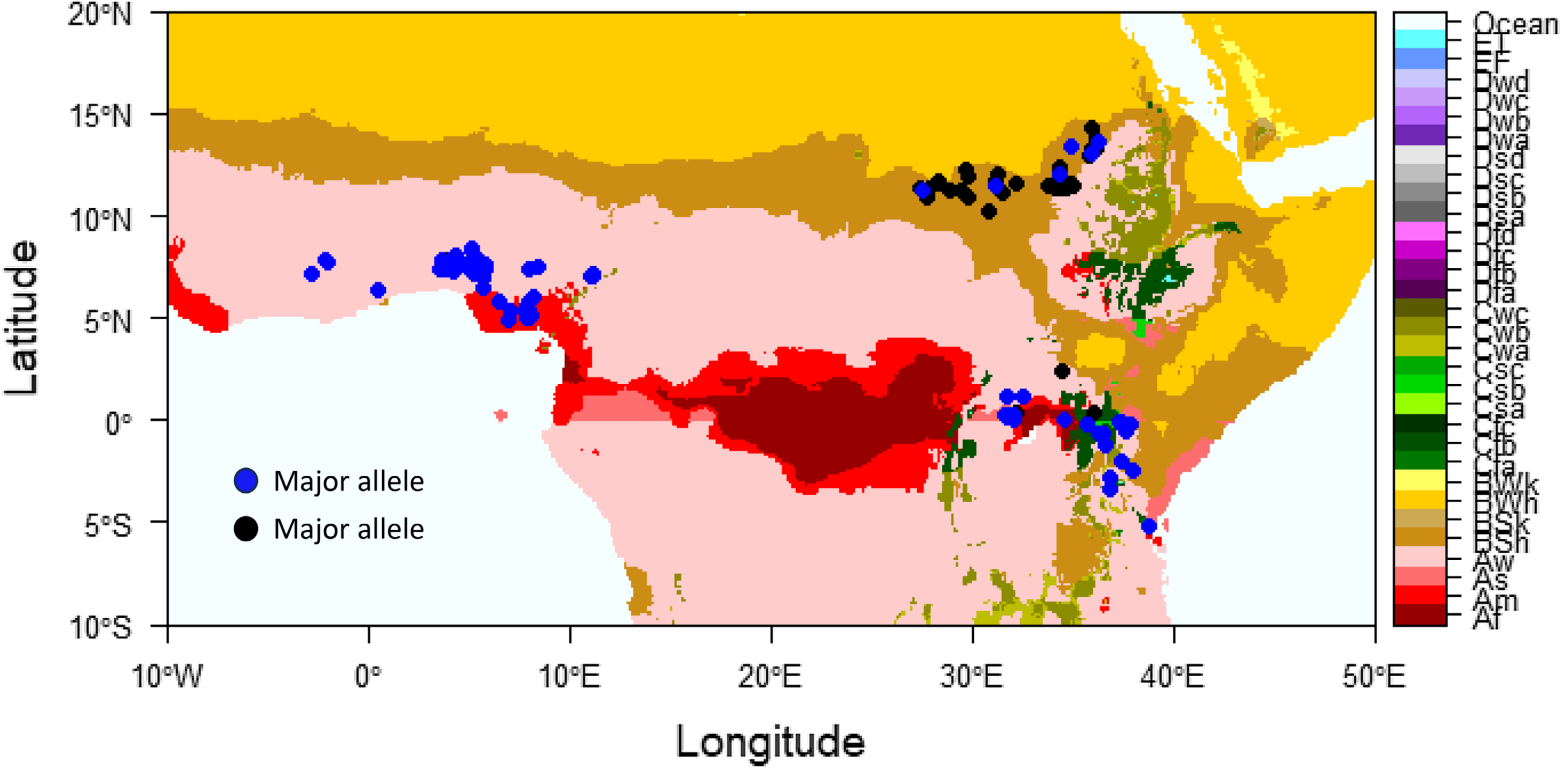
The sampling points and allele frequency distribution of a candidate SNP Chr8_71590031 detected by partial RDA linked to protein AKT1 expressed in response to water deprivation and salt stress and regulates potassium ion transport and stomatal closure. The colors represent the Köppen climate classification (Detailed description in Table S8). The West African sampling climates include tropical monsoons and tropical savannas with dry winters. East African environments consist of Sudan's dry, semi‐arid hot climates (BSh) and Kenyan and Ugandan tropical and temperate climates (Af, As, Aw, BSh, and Cfb). The first letters in the climate classification acronyms represent A—tropical climate; B—Arid climate; C—Temperate climate; D—cold continental climate; and E—Polar climate.

**FIGURE 5:**
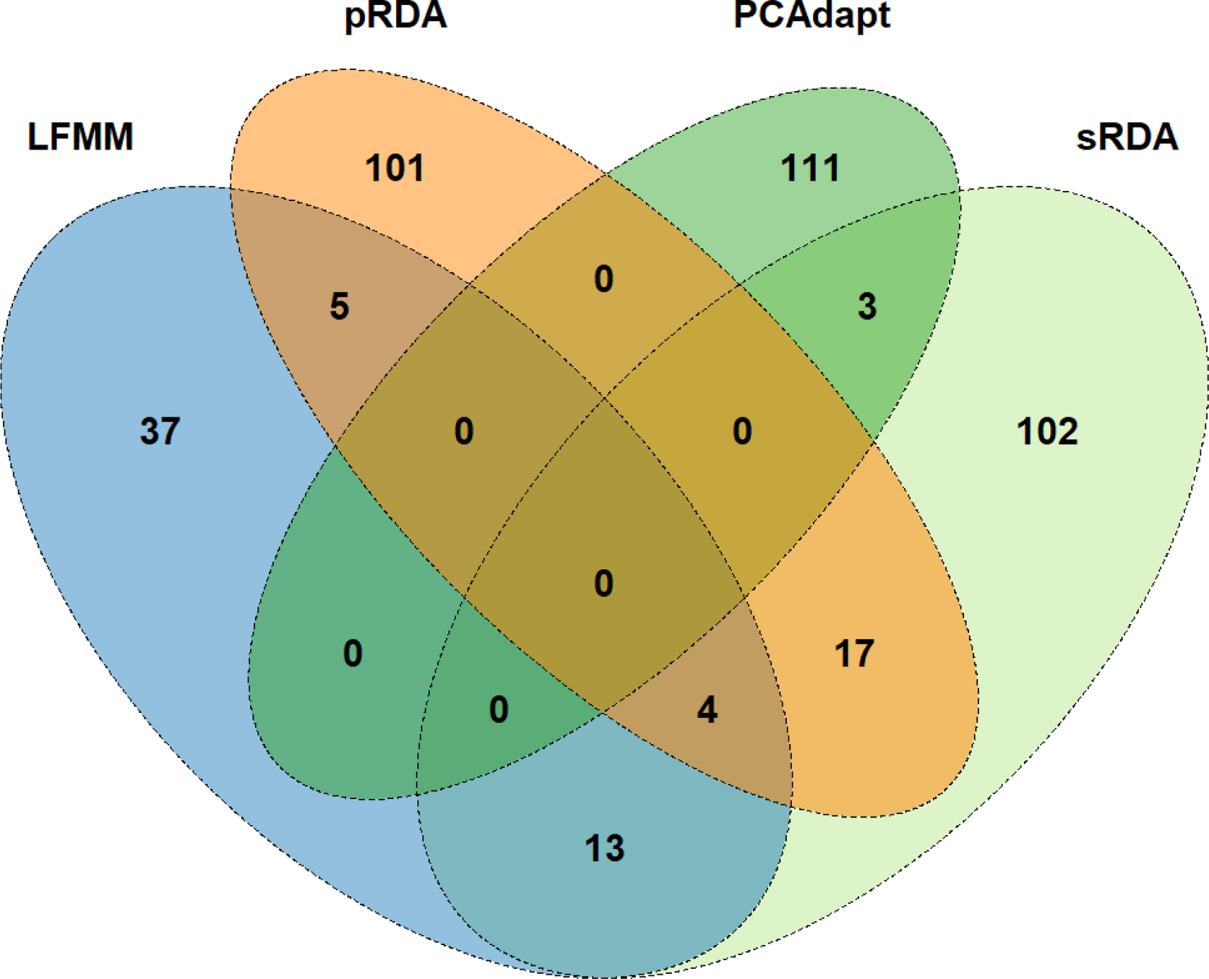
A Venn diagram showing the number of significant SNPs detected by four genome scan methods.

In summary, we detected 443 candidate SNPs using the four methods (Figure 4; Table S10). Fifty SNP among the total detections were duplicated among the selection methods. The candidate SNPs were distributed across all 12 chromosomes, as shown in the Manhattan plots (Figures S6 and S7). Most SNPs were associated with soil nitrogen content (104), mean temperature of the wettest quarter (64), and wind (63). The simple RDA, partial RDA, LFMM, and PCAdapt identified 139, 127, 63, and 114 candidate SNPs, respectively (Figure 5; Table S6). Despite a minimal overlap of SNPs identified by the method, 42 SNPs were identified by at least two methods. None was identified by more than three methods (Figure 5).

Approximately 40 of the 393 unique candidate SNPs (Table S9) were directly associated with candidate genes contributing to adaptation to different abiotic stressors such as heat, cold, drought, and salinity in the Uniprot and TAIR databases. For example, SNP Chr8_71590031 (Table 3), located in Chromosome 8, is associated with nitrogen content in the soil and is linked to a protein *AKT1* (characterized in *Arabidopsis thaliana*). This protein regulates stomatal closure and root elongation and responds to water deprivation and salt stress (Nieves‐Cordones et al., 2012; Pyo et al., 2010). Sixty‐five of the detected candidate SNPs were mapped within genes for proteins of unknown functions.

**TABLE 3:** Forty‐two candidate SNPs detected by more than one selection test, the associated environmental variables, gene designation, and the descriptions of the gene functions.

SNP_ID	Env. Variable	Gene/protein	Function
Chr0_35225179	Wind, cmi_range	AtMg01280	Involved in ATP synthesis coupled electron transport^19^
Chr1_21727588	cec	Os02g0194200	Regulation of growth, development, and stress adaptation in plants^6^
Chr1_9302370	MTWeQ	R1A	Plant‐type hypersensitive response^5^
Chr2_67878080	Nitrogen	PSY1	Chloroplast organization; regulation of cell population proliferation^20^
Chr2_67878140	Nitrogen	PSY1	Chloroplast organization; regulation of cell population proliferation^20^
Chr3_70109310	cec	At3g06530	Positive regulation of transcription by RNA polymerase^21^
Chr4_60263700	Nitrogen	ASHH2	Response to nitrate starvation; negative regulation of flower development^22^
Chr4_60263765	Nitrogen	ASHH2	Response to nitrate starvation; negative regulation of flower development^22^
Chr4_60263581	Nitrogen	ASHH2	Response to nitrate starvation; negative regulation of flower development^22^
Chr4_60263643	Nitrogen	ASHH2	Response to nitrate starvation; negative regulation of flower development^22^
Chr4_64533143	MTWeQ	At1g21400	Response to absence of light; cellular response to sucrose starvation^25^
Chr5_9075140	MTWeQ, silt	SD11	Protein phosphorylation; pollen recognition^26^
Chr5_9075023	MTWeQ, silt	SD11	Protein phosphorylation; pollen recognition^26^
Chr5_72629041	MTWeQ	NAC017	Leaf senescence^28^
Chr5_72628953	MTWeQ	NAC017	Leaf senescence^28^
Chr6_85268807	MTWeQ	Unknown	Protein of unknown function
Chr6_96411126	MTWeQ	Unknown	Protein of unknown function
Chr6_96581016	Nitrogen	TIR1	Auxin‐activated signaling pathway; stamen development; lateral root formation^30^
Chr7_104219631	Nitrogen	FLXL1	Involved in cell differentiation and flower development^1^
Chr8_1265436	Nitrogen	Unknown	Protein of unknown function
Chr8_34572924	Silt	Unknown	Protein of unknown function
Chr8_71590031	Nitrogen	AKT1	Potassium ion import, regulate stomatal closure, response to salt stress, water deprivation, and root hair elongation^2,3^
Chr8_85565240	MTWeQ	CYP80G2	Secondary metabolite biosynthetic process^4^
Chr9_1078596	Silt, cec	At4g18465	May be involved in pre‐mRNA splicing^5^
Chr9_18066040	Wind	HCR9‐0	Response to fungal pathogen^7^
Chr9_19520528	Nitrogen, wind	Unknown	Protein of unknown function
Chr9_46071667	Nitrogen	TIC110	Involved in protein precursor import into chloroplasts^8^
Chr9_46071601	Nitrogen	TIC110	Involved in protein precursor import into chloroplasts^8^
Chr9_81545381	Nitrogen	CIA1	Iron–sulfur cluster assembly^9^
Chr9_73670827		CIA1	Iron–sulfur cluster assembly^10^
Chr9_84539405	MTWeQ	ZIP5	Mediates zinc uptake from the rhizosphere^11^
Chr9_84539456	MTWeQ	ZIP5	Mediates zinc uptake from the rhizosphere^11^
Chr9_84539273	MTWeQ	ZIP5	Mediates zinc uptake from the rhizosphere^11^
Chr9_91043940	Nitrogen	At5g18390	Post‐transcriptional regulations of nuclear genes^31^
Chr10_1140024	cec	CP2	Participates in the epigenetic repression of flowering genes^11,13^
Chr10_62134775	pet_range, wind	CYP714A1	Involved in the inactivation of early gibberellin (GA) intermediates^14^
Chr10_73179139	Nitrogen	CPN60B4	Involved in protein folding^5^
Chr10_73179037	Nitrogen	CPN60B4	Involved in protein folding^5^
Chr10_82319771	Wind	Unknown	Protein of unknown function
Chr11_162162	PDrM	Unknown	Protein of unknown function
Chr11_30958401	Wind	ATRX	Maintenance of rDNA; pollen development^17^
Chr11_96396192	Nitrogen	MYOB2	Myosin XI tail binding^18^

*Note*: ^1^Lee & Amasino, 2013; ^2^Nieves‐Cordones et al., 2012; ^3^Pyo et al., 2010; ^4^Ikezawa et al., 2008; ^5^The UniProt Consortium, 2023; ^6^Han et al., 2021; ^7^Parniske et al., 1997; ^8^Yuan et al., 2021; ^9,10^Luo et al., 2012; ^11^Lee et al., 2010; ^13^Nadi et al., 2018; ^14^Zhang et al., 2011; ^17^Duc et al., 2017; ^18^Kurth et al., 2017; ^19^Zsigmond et al., 2008; ^20^Ruckle et al., 2012; ^21^Gaudet et al., 2011; ^22,23,24^Li et al., 2023; ^25^Fujiki et al., 2001; ^26^Samuel et al., 2008; ^28^Meng et al., 2019; ^30^Yu et al., 2015; ^31^Colcombet et al., 2013.

We illustrated the allele frequency distribution of the SNP Chr8_71590031 selected based on its link to a protein with an obvious function in adaptation to drought and salt stress (Figure 5). We observed a clear difference in the allele frequency distribution along the environmental gradient associated with different climates and soil nitrogen content. The allele distribution patterns are distinct between Sudan's dry, semi‐arid, hot regions, where the minor alleles are observed, and the tropical monsoon and dry winter savannas of Nigeria and Ghana, where the major alleles dominate.

### 3.5 Adaptive landscape

**FIGURE 6:**
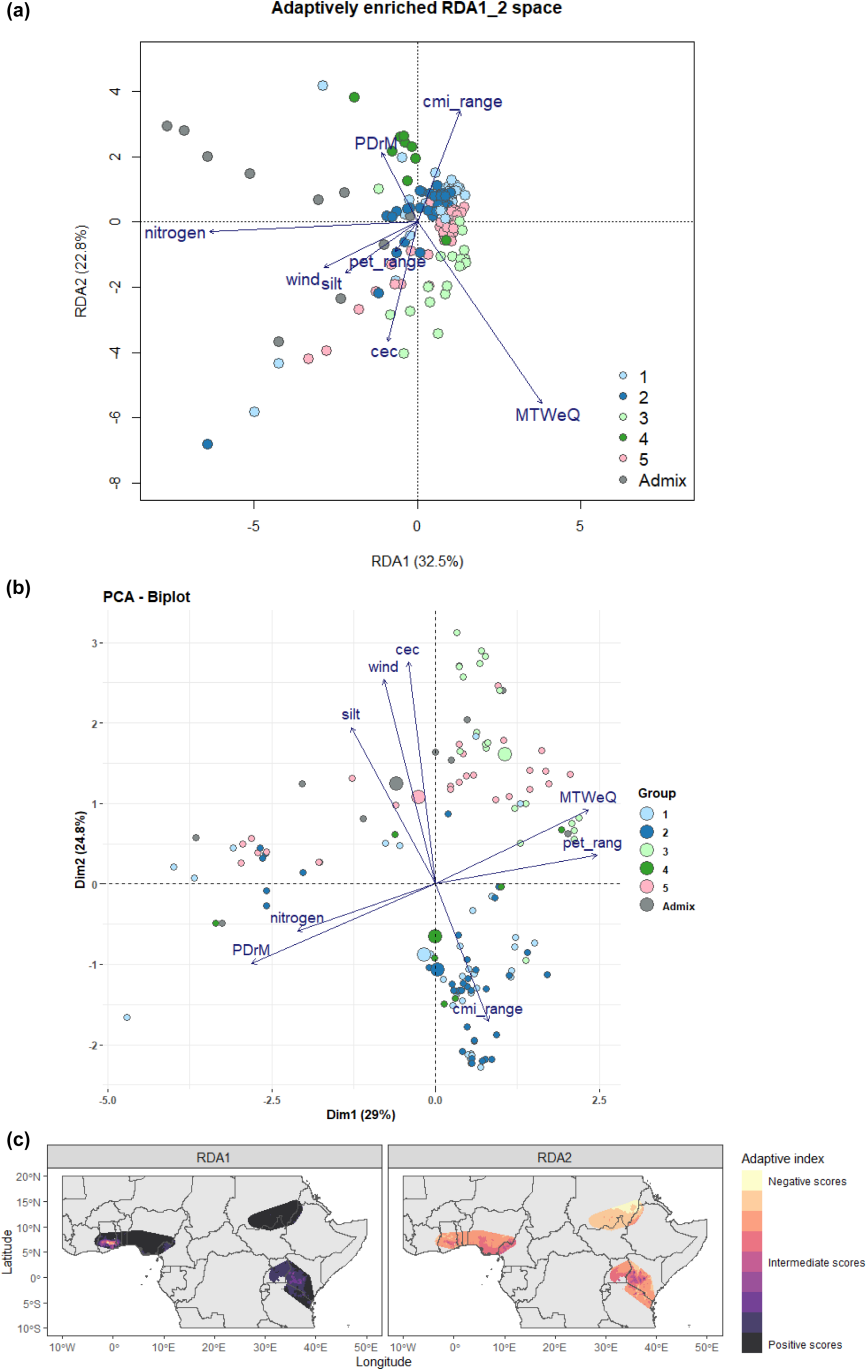
Adaptive landscape with (a) the adaptively enriched genetic space showing the association between regional sampling locations and environmental drivers of adaptation; (b) Principal component analysis of the environmental diversity according to the sampling regions. The colors in a and b represent the groups detected in the population structure analysis. (c) the spatial projection of the adaptability across the study areas. PDrM: Precipitation of the driest quarter; MTWeQ: Mean temperature of the wettest quarter; wind: wind speed; cmi_range: Annual range of monthly climate moisture index; pet_range: Annual range of potential evapotranspiration; nitrogen: Soil nitrogen content; silt: Soil silt content; cec: Soil cation exchange capacity.

The modeled environmental space enriched with candidate SNPs is illustrated in Figure 6. The RDA performed purely with the outlier SNPs revealed a separation of the samples in the second axis according to the environmental characteristics of the sampling sites and according to the groups detected in the population structure analysis. This observation further strengthens the higher contribution of the environment in explaining the population structure. A PCA of the selected environmental variables could also detect the groups identified by population structure analysis (Figure 6b). The clustering of the groups in the environmental PCA largely mirrored the collection regions. Groups 1 and 2 consist mainly of samples from West Africa, separated from the rest of the groups with East African origin samples. This indicates that the set of environmental variables and the selected SNPs can be effectively used to characterize the environments and adaptive genetic diversity of the African eggplant wild relatives. The allele frequencies along the first RDA axis (32.5%) were primarily associated with soil nitrogen content, mean temperature of the wettest quarter, and wind. The second RDA axis (22.8%), on the other hand, was mainly associated with the mean temperature of the wettest quarter soil cation exchange capacity and Annual range of monthly climate moisture index (cmi_range) (Figure 6a). When our adaptive landscape model was projected geographically, the first RDA axis contrasted the West African (Ghana) and East African (Kenya) from the rest of the regions. The East African (Kenyan) and West African (Ghanaian) populations experience relatively higher soil nitrogen content, the main driver in the first RDA axis. Our adaptive landscape on the second RDA axis also contrasted East Africa (Sudan), which is experiencing arid and semi‐arid climates characterized by higher temperatures compared to the tropical and temperate climates of West Africa and East Africa.

To estimate the adaptive potential of the species, we calculated the adaptive index as described in Methods. The species average of the adaptive scores are shown in Table S8. On the first RDA Axis, *Solanum anomalum* and *S. macrocarpon s*howed, on average, the highest positive adaptive scores, mainly related to the West African environments with high climate moisture index, low wind speeds, and soils with low cation exchange capacity and silt content. *Solanum nigriviolecuem* and *S. mauense* showed the most negative adaptive scores on the first RDA axis, mainly related to an environment with low temperatures in the wettest quarter, high precipitations in the driest month, and high soil nitrogen contents. Other species, including *S. aculeastrum*, *S. aculeatissimum*, and *S. arundo*, sampled from similar environments, generally showed low adaptive scores on the first RDA axis. *S. anguivi* showed a high range of adaptive scores in the first due to the environmental differences of their sampling sites. This shows the environmental influence even within species (Table S7). On the second RDA axis, *S. mauense*, *S. aculeastrum*, and *S. nigriviolaceum* showed the highest positive adaptive scores compared to other species, contrasting with the observation in the first axis. *Solanum coagulans* and *S. incanum* showed the most negative adaptive scores on the second RDA axis, mainly related to an environment with high soil nitrogen content, highest temperatures in the wettest quarter, lowest precipitation in the driest month, high potential evapotranspiration ranges and soils with the highest cation exchange capacity.

## 4 DISCUSSION

### 4.1 The genetic variation across eggplant crop wild relatives significantly corresponds to environmental adaptation

Our findings demonstrate a significant correlation between genetic variation and environmental differences in the wild eggplant species, even though the environment does not account for the highest proportion of explainable genetic variation. Overall, genetic differentiation was found at high levels among the groups representing accessions from different environments. The results of the UPGMA tree and population structure analysis also confirm this. These findings provide insights into to which extent populations of wild eggplant relatives have been shaped by environmental adaptation. Even though the speciation is beyond the scope of this study, our results might also highlight the environment's role in speciation in the eggplant gene pools, which is in line with previous reports on eggplant species divergence (Weese & Bohs, 2010).

The RDA analysis showed that climate and soil are important environmental drivers of genomic variations in our materials. Including a range of environmental data in landscape genomics studies will help effectively evaluate the complex factors contributing to local adaptation in nature (Dauphin et al., 2023). So far, few landscape genomic studies have integrated soil factors such as pH and nitrogen in the analysis. Even though the climate may influence soil development (Joswig et al., 2022), our findings highlight the importance of including soil factors in landscape genomics as drivers of environmental adaptation. This is also in line with other studies. For example, a study on the adaptation of two grasshopper species in the Australian Alps revealed significant GEAs with soil pH, among other factors (Yadav et al., 2021). Arabidopsis demes were also found to be locally adapted in their native habitat to soils with moderately high carbonate (Terés et al., 2019).

Our study observed a significant effect of the geographic distances between the sampling sites compared to the environmental factors. This aligns with other studies for the plant species *Boechera stricta* genotypes from populations experiencing different climatic conditions (Chang et al., 2022; Cruz‐Nicolás et al., 2020; Malanson et al., 2017). In other studies, geographical distance and environmental factors explained comparable proportions of the genetic variation (Gibson & Moyle, 2020; Lasky et al., 2012, 2015). Thus, the proportional effects of environment and geography depend heavily on the species, its environment, and the species' history in that environment.

We found a relatively low proportion of the explainable SNP variation due to environmental (6.0%). This appears to be common in other landscape studies that report a low percentage of variation explained by the environment (Dauphin et al., 2023). Several factors we did not address in our study may be responsible for this phenomenon. Firstly, despite incorporating several environmental variables in this study, other evolutionary forces, such as pests and diseases unrelated to the factors used in our study, may also play a role. Detecting several SNP in genes conferring immunity and resistance against insects in this study might support this claim. Also, while our environmental data provide information at a larger geographic scale, they may not necessarily capture all local environmental heterogeneity. Another reason for low explainable SNP variation may arise because RDA and LFMM can only model linear associations between the environment/space and SNPs and, therefore, will fail to capture non‐linear associations that might exist (Borcard et al., 2011). Nevertheless, RDA models remain effective because they can effectively account for covariation among environmental variables and genetic markers, as is often the case in nature (Capblancq & Forester, 2021).

Our findings show that the eggplant's West and East African populations exhibited distinctive genetic responses due to climatic and soil factors. The first adaptive component was associated with soil nitrogen content and the mean temperature in the wettest quarter, while the second component was associated with the mean temperature of the wettest quarter, climate moisture index, and cation exchange capacity of the soil. This component contrasts the West African sampling areas with the East African sampling areas. These results suggest that the populations respond to selective gradients related to soil quality, temperature, and water availability. We also get the impression that the covariation between soil and climate is oversimplified, so it would be important to investigate the effect of the two in driving selection. Identifying species with interesting traits is paramount to effectively using CWR diversity. The description of the adaptive landscape enables the identification of putatively adapted genotypes returning high adaptive scores. In this study, *S. anomalum*, *S. macrocarpon*, *S. incanum*, and *S. coagulans* showed the highest adaptive scores of 1.02, 1.01, 0.42, and 0.42, respectively. The high adaptive score for these species, especially *S. incanum*, affirms records that they are known to grow in desert conditions and, especially *S. incanum*, is considered a powerful source of phenolics and tolerant to abiotic stress such as drought (Gramazio et al., 2017; Knapp et al., 2013; Meyer et al., 2012; Plazas et al., 2022).

Our population structure analysis revealed admixture in all the species in our study, suggesting gene flow and interspecific hybridization occur regularly among eggplant wild relatives. The interfertile nature of the eggplant species can explain the admixtures and clustering of the species from different *Solanum* clades. Interspecific hybridization between eggplant species has been shown mostly in crossing experiments (Bukenya & Carasco, 1995; Plazas et al., 2016) and between domesticated eggplants and their wild ancestors (Meyer et al., 2012). However, we have not found reports so far on the interspecific hybridization among eggplant wild relatives in their natural environment. This provides evolutionary insights, has implications for in situ conservation, and requires further investigation to understand these natural patterns of interspecific hybridization. These findings also confirm the relevance of carrying out landscape genomics for crop wild relatives at the gene poolgene pool level rather than individual species, as the gene pools of eggplant and many other crops, such as those of pumpkin and amaranth have significant levels of interspecific fertility (Lin et al., 2022; Sanjur et al., 2002).

One limitation of our study was the seemingly biased sampling of species in the different regions involved in our study. This is mainly due to the rare record of some of the species. Furthermore, as much as crop wild relatives are known to thrive in diverse marginal environments, some species may be limited to particular environments (Renzi et al., 2022). Expanding this study with new collections will provide further information about environmental adaptation. However, our current study intends to fully utilize the extensive untapped genetic diversity in eggplant genebank material by incorporating rare species from the same gene pools as the more common species. Many accessions of eggplant and related species from primary, secondary, and tertiary gene pools have not been thoroughly assessed despite possessing diverse traits beneficial for eggplant breeding, including traits adaptive to climate change (Gramazio et al., 2023). Consequently, our study offers valuable insights that can guide breeders and conservation experts on important adaptive characteristics that might be overlooked if rare species are disregarded.

Because the adaptive landscape is associated with environmental factors, conservation managers may also apply our findings to effectively manage populations experiencing different ecological conditions and facilitate future studies on eggplant populations' response to future environments. Several tools and methodologies have been developed to predict changes in allele frequencies under climate change to support in situ conservation and germplasm collecting (Dauphin et al., 2023; Rellstab et al., 2016). What our study shows is that this type of climate assessment can be extended to look at evolutionary patterns at a larger genepool or clade rather than assessments for single taxa. This allows a broader view on the conservation of genomic variation and also allows the inclusion of rare taxa that have unique genomic variation compared to other taxa.

### 4.2 Landscape genomics detected candidate SNP markers for both climate and soil factors

Our outlier detection approaches identified 396 candidate SNP markers—less than one percent of the total number of SNPs of the initial selection. Other studies have also observed percentages similar to ours (Chang et al., 2022; Mdladla et al., 2018). We attribute this to several reasons. Likely, more loci are under selection, but the stringency applied in our analysis did not allow their detection. The control for population structure in GEA methods can sometimes be overly conservative (Forester et al., 2018). Therefore, we applied multiple methods in our study to capture more candidate SNPs. We attribute their complementarity to the four methods' different assumptions, strengths, and limitations. At the same time, the conservative selection reduces the number of false positives, and a large proportion of the final set of candidate SNPs could be associated with candidate adaptive genes that have been reported. Secondly, adaptation occurs mostly due to minor and linked allele frequency modifications across multiple genetic loci that show weak selection and may not be detected through the genome scan (Stetter et al., 2018). Finally, the GBS method we applied in generating our SNPs only allows the examination of low coverage of the genomes, thereby limiting the total number of SNPs used in our analysis and the ability to detect a considerable number of adaptive candidate genes. Future whole‐genome studies will allow the detection of more candidate SNP markers.

The three hundred and ninety‐three outliers identified by our detection methods revealed a pattern of environmental separation in our populations (Figure 6a). This finding suggests that these markers are highly linked to selection at the natural sites. Among the SNP identified, we considered the 42 retained by more than one detection method to constitute a strong signal (Hoban et al., 2016), some of which are located in genes associated with plant adaptation and have been characterized in other plant species, including *Arabidopsis thaliana*, *Solanum lycopersicum*, *Solanum demissum*, and *Capsicum annuum*. These genes may be prioritized for further functional analysis in eggplant. Among the most interesting are those that act to regulate abiotic stress tolerance. SNP Chr8_71590031, for instance, is located in gene *AKT1*, acts in response to salt and water stress, and is also involved in regulating stomatal closure, root hair elongation, and potassium ion transport. Disruption of protein *AKT1* in *Arabidopsis thaliana* conferred an enhanced response to water stress where adult plants lost less water than the wild‐type (Nieves‐Cordones et al., 2012). Another is the CCCH zinc‐finger protein, *Os02g0194200*, in *Oryza sativa* subsp. Japonica. This protein was associated with the soil cationic exchange capacity. CCCH‐type finger proteins have been widely associated with function related to the adaptation of plants to multiple abiotic stresses in various ways described in a review by Han et al., 2021. In rice, for instance, ABA‐induced CCCH zinc‐finger protein reduces sensitivity to ABA to enhance drought tolerance (Wang et al., 2015).

We also identified genes regulating plant growth, reproductive development, and transcription. Among the genes we detected were also genes involved in flower development. For instance, protein *FLXL1* is part of FRIGIDA complex that acts as a molecular switch underlying the activation of FLOWERING LOCUS (FLC) for flowering time control in *A. thaliana*. Protein *ASHH2*, a histone methyltransferase, also positively regulates FLC to prevent early flowering transition (Zhao et al., 2005). Double mutant of the *CP2* gene in *A. thaliana* has also been shown to skip vegetative development and flower upon germination (Mateo‐Bonmatí et al., 2005). Also, *Cpn60β4* mutant plants showed early seed germination and early flowering phenotype in *A. thaliana* (Tiwari & Grover, 2019). The timing of flowering is an important adaptive trait to prevent time to enable reproductive success. From the adaptation perspective, suppressing or accelerating the transition from vegetative to reproductive development in plants shows clear fitness consequences in harsh environments (Austen et al., 2017; Li et al., 2010; Shafiq et al., 2014). Flowering too early or too late can increase the chances of floral damage and the risk of incomplete seed development due to adverse weather conditions (Inouye, 2008). Nitrogen is a dominant macronutrient affecting flowering time and general plant growth (Zhang et al., 2022); therefore, it is not surprising that all the genes regulating flowering time in this study were associated with soil nitrogen content.

Protein *AKT1* is also involved in root development. This protein, correlated to soil nitrogen content, shows a directional change in allele frequency to soil nitrogen content. We have also demonstrated in this study how minor allele frequency was observed predominantly in the Sudan population experiencing hot and dry climates where nitrogen availability is limited (Peri et al., 2019). Under nitrogen deficiency, roots show root‐related differential expression of proteins contributing to enhanced root growth (Qin et al., 2019). These findings offer valuable insights for investigating eggplant root response to nitrogen deficiency and the development of cultivars with high nitrogen use efficiency through genetic improvements.

The multiple associations of soil nitrogen content with candidate SNPs from gene functions related to root nodulation and bacterial and fungal infections could also suggest that soil microbiomes have an important role in the environmental adaptation of eggplant crop wild relatives. Recent research is starting to unravel the role of these microbiomes in plant adaptation to environmental stress (AL‐surhanee et al., 2021; Pasbani et al., 2020). Including microbiome diversity in landscape genomics could provide further insights into the environmental adaptation of plant species.

Other promising candidate genes include Zinc transporter 5 (*ZIP5*), involved in Zinc ion transport (Lee et al., 2010); *NAC017* protein, a transcription factor key in regulating mitochondrial proteotoxic stress responses in plants (Kacprzak et al., 2020); *CYP714A1* protein involved in the inactivation of gibberellin intermediates (Zhang et al., 2011); protein *ATRX* involved in the maintenance of the rDNA and pollen development (Duc et al., 2017); *TIC110* protein facilitating plastid pre‐protein translocation (Yuan et al., 2021) and *CIA1* protein essential component of the cytosolic iron–sulfur (Fe‐S) protein assembly (*CIA*) machinery (Luo et al., 2012). A few other candidates we detected have unknown functions would make good candidates for further investigations.

While our study detected just a few candidate genes conferring environmental adaptation, we imagine these genes could be linked to other genes in gene clusters that drive traits involved in environmental adaptation. Therefore, these genes can be useful in gene co‐expression network analysis to map other genes that could contribute to adaptation traits.

This is the first study identifying the candidate SNPs to detect adaptive genes in eggplant wild relatives. Our set of candidate SNP markers provides a new genomic tool for eggplant breeding, and our study provides an example of how landscape genomics can be applied to crop wild relatives. Our analysis thus provides a toolbox for breeders and researchers to establish new phenotyping experiments to test specific relations between genes and the environment. Associating the candidate SNP markers to environmental factors is a primary step in uncovering the adaptive process. However, these correlations do not necessarily confirm the occurrence of environmental adaptation in nature. Therefore, the next step is to validate these genes with functional analysis in transcriptome gene expression analysis.

## 5 CONCLUSIONS

Overall, this study has successfully analyzed the association between the environmental factors and the genetic markers to determine the effect of the environmental factors on the explainable genetic variation. Even though climate influences soil formation, we observed that soil factors play a prominent role as key explanatory aspects of genetic variation. The GEA and outliers tests identified genomic regions that might contribute to local adaptation in eggplant wild relatives. One limitation is the sparse identification of outlier SNP that might have been occasioned by the GBS approach of sequencing only a small portion of the genome. The correlations between the environmental factors and the genes we have detected in this study help to understand the natural selection of eggplant wild relatives. However, common garden experiments, transplantation experiments, and gene expression studies of differentially adapted genotypes will help provide stronger evidence for fitness in different environments. Taken together, our study provides valuable insights into the environmental adaptation of eggplant CWR with clear applications for breeders and conservation managers. We showed that environmental selection is a critical factor in the genetic variation within a crop's wild relatives, which is widespread and diverse due to climate and soil factors.

## AUTHOR CONTRIBUTIONS

**Emmanuel O. Omondi:** Conceptualization (lead); data curation (lead); formal analysis (lead); methodology (lead); visualization (lead); writing – original draft (lead); writing – review and editing (lead). **Chen‐Yu Lin:** Methodology (supporting); writing – review and editing (supporting). **Shu‐Mei Huang:** Methodology (supporting); writing – review and editing (supporting). **Cheng‐An Liao:** Methodology (supporting); writing – review and editing (supporting). **Ya‐Ping Lin:** Methodology (supporting); writing – original draft (supporting); writing – review and editing (equal). **Ricardo Oliva:** Writing – original draft (supporting); writing – review and editing (equal). **Maarten van Zonneveld:** Conceptualization (lead); formal analysis (supporting); funding acquisition (lead); investigation (supporting); methodology (supporting); project administration (lead); resources (lead); supervision (lead); writing – original draft (supporting); writing – review and editing (lead).

## CONFLICT OF INTEREST STATEMENT

The authors declare that they have no conflict of interest.

## Supporting information


Figure S1.

Table S1.

## Data Availability

Sample descriptions and environmental variables are provided as supporting materials. The SNP dataset is available on Dryad (https://datadryad.org/stash/share/QF9UI_3NOWOwwy8S3UINq0qXMwzbVWTBO2uvMWsGvz0).

## References

[ece311662-bib-0001] AL‐surhanee, A. A. , Soliman, M. H. , & Ouf, S. A. (2021). The role of soil microbes in the plant adaptation to stresses: Current scenario and future perspective. Frontiers in Plant‐Soil Interaction, 237–258. 10.1016/B978-0-323-90943-3.00006-7

[ece311662-bib-0002] Aubriot, X. , Knapp, S. , Syfert, M. M. , Poczai, P. , & Buerki, S. (2018). Shedding new light on the origin and spread of the brinjal eggplant (*Solanum melongena* L.) and its wild relatives. American Journal of Botany, 105(7), 1175–1187. 10.1002/ajb2.1133 30091787

[ece311662-bib-0003] Aubriot, X. , Singh, P. , & Knapp, S. (2016). Tropical Asian species show that the Old World clade of ‘spiny solanums’ (Solanum subgenus Leptostemonum pro parte: Solanaceae) is not monophyletic. Botanical Journal of the Linnean Society, 181(2), 199–223. 10.1111/boj.12412

[ece311662-bib-0004] Austen, E. J. , Rowe, L. , Stinchcombe, J. R. , & Forrest, J. R. K. (2017). Explaining the apparent paradox of persistent selection for early flowering. New Phytologist, 215(3), 929–934. 10.1111/nph.14580 28418161

[ece311662-bib-0005] Barchi, L. , Rabanus‐Wallace, M. T. , Prohens, J. , Toppino, L. , Padmarasu, S. , Portis, E. , Rotino, G. L. , Stein, N. , Lanteri, S. , & Giuliano, G. (2021). Improved genome assembly and pan‐genome provide key insights into eggplant domestication and breeding. The Plant Journal, 107, 579–596. 10.1111/tpj.15313 33964091 PMC8453987

[ece311662-bib-0006] Beck, H. , Zimmermann, N. , McVicar, T. , Vergopolan, N. , Berg, A. , & Wood, E. F. (2018). Present and future Köppen‐Geiger climate classification maps at 1‐km resolution. Scientific Data, 5, 180214. 10.1038/sdata.2018.214 30375988 PMC6207062

[ece311662-bib-0007] Becker, O. , Minka, A. , & Deckmyn, A. (2022). maps: Draw geographical Maps . R Package Version 3.4.1. https://CRAN.R‐project.org/package=maps

[ece311662-bib-0008] Bianchini, G. , & Sánchez‐Baracaldo, P. (2024). TreeViewer: Flexible, modular software to visualise and manipulate phylogenetic trees. Ecology and Evolution, 14(2), e10873. 10.1002/ece3.10873 38314311 PMC10834882

[ece311662-bib-0009] Borcard, D. , Gillet, F. , & Legendre, P. (2011). Numerical ecology with R. Springer International Publishing AG.

[ece311662-bib-0010] Bradbury, P. J. , Zhang, Z. , Kroon, D. E. , Casstevens, T. M. , Ramdoss, Y. , & Buckler, E. S. (2007). TASSEL: Software for association mapping of complex traits in diverse samples. Bioinformatics, 23(19), 2633–2635. 10.1093/bioinformatics/btm308 17586829

[ece311662-bib-0011] Brun, P. , Zimmermann, N. E. , Hari, C. , Pellisier, L. , & Karger, D. N. (2022). Global climate‐related predictors at kilometre resolution for the past and future. Earth System Science Data, 14, 5573–5603. 10.5194/essd-14-5573-2022

[ece311662-bib-0012] Bukenya, Z. R. , & Carasco, J. F. (1995). Crossability and cytological studies in *Solanum macrocarpon* and *Solanum linnaeanum* (Solanaceae). Euphytica, 86, 5–13. 10.1007/BF00035933

[ece311662-bib-0013] Capblancq, T. , & Forester, B. R. (2021). Redundancy analysis: A Swiss Army knife for landscape genomics. Methods in Ecology and Evolution, 12(12), 2298–2309. 10.1111/2041-210X.13722

[ece311662-bib-0014] Capblancq, T. , Luu, K. , Blum, M. G. B. , & Bazin, E. (2018). Evaluation of redundancy analysis to identify signatures of local adaptation. Molecular Ecology Resources, 18, 1223–1233. 10.1111/1755-0998.12906 29802785

[ece311662-bib-0015] Catchen, J. , Hohenlohe, P. A. , Bassham, S. , Amores, A. , & Cresko, W. A. (2013). Stacks: An analysis tool set for population genomics. Molecular Ecology, 22(11), 3124–3140. 10.1111/mec.12354 23701397 PMC3936987

[ece311662-bib-0016] Caye, K. , Jumentier, B. , Lepeule, J. , & François, O. (2019). LFMM 2: Fast and accurate inference of gene‐environment associations in genome‐wide studies. Molecular Biology and Evolution, 36(4), 852–860. 10.1093/molbev/msz008 30657943 PMC6659841

[ece311662-bib-0017] Chang, C. W. , Fridman, E. , Mascher, M. , Himmelbach, A. , & Schmid, K. (2022). Physical geography, isolation by distance, and environmental variables shape the genomic variation of wild barley (*Hordeum vulgare* L. ssp. *spontaneum*) in the southern levant. Heredity, 128, 107–119. 10.1038/s41437-021-00494-x 35017679 PMC8814169

[ece311662-bib-0018] Chitwood‐Brown, J. , Vallad, G. E. , Lee, T. G. , & Hutton, S. F. (2021). Characterization and elimination of linkage‐drag associated with fusarium wilt race 3 resistance genes. Theoretical and Applied Genetics, 134(7), 2129–2140. 10.1007/s00122-021-03810-5 33786652 PMC8263443

[ece311662-bib-0019] Colcombet, J. , Lopez‐Obando, M. , Heurtevin, L. , Bernard, C. , Martin, K. , Berthomé, R. , & Lurin, C. (2013). Systematic study of subcellular localization of *Arabidopsis* PPR proteins confirms a massive targeting to organelles. RNA Biology, 10(9), 1557–1575. 10.4161/rna.26128 24037373 PMC3858439

[ece311662-bib-0020] Cruz‐Nicolás, J. , Giles‐Pérez, G. , González‐Linares, E. , Múgica‐Gallart, J. , Lira‐Noriega, A. , Gernandt, D. S. , Eguiarte, L. E. , & Jaramillo‐Correa, J. P. (2020). Contrasting evolutionary processes drive morphological and genetic differentiation in a subtropical fir (*Abies*, Pinaceae) species complex. Botanical Journal of the Linnean Society, 192(2), 401–420. 10.1093/botlinnean/boz077

[ece311662-bib-0021] Dauphin, B. , Rellstab, C. , Wüest, R. O. , Karger, D. N. , Holderegger, R. , Gugerli, F. , & Manel, S. (2023). Re‐thinking the environment in landscape genomics. Trends in Ecology & Evolution, 38(3), 261–274. 10.1016/j.tree.2022.10.010 36402651

[ece311662-bib-0022] Dempewolf, H. , Baute, G. , Anderson, J. , Kilian, B. , Smith, C. , & Guarino, L. (2017). Past and future use of wild relatives in crop breeding. Crop Science, 57, 1070–1082. 10.2135/cropsci2016.10.0885

[ece311662-bib-0023] Dempewolf, H. , Eastwood, R. J. , Guarino, L. , Khoury, C. K. , Müller, J. V. , & Toll, J. (2014). Adapting agriculture to climate change: A global initiative to collect, conserve, and use crop wild relatives. Agroecology and Sustainable Food Systems, 38(4), 369–377. 10.1080/21683565.2013.870629

[ece311662-bib-0024] Dray, S. , Bauman, D. , Blanchet, G. , Borcard, D. , Clappe, S. , Guenard, G. , Jombart, T. , Larocque, G. , Legendre, P. , Madi, N. , & Wagner, H. H. (2022). adespatial: Multivariate multiscale spatial analysis . R Package Version 0.3‐7. https://CRAN.R‐project.org/package=adespatial

[ece311662-bib-0025] Dray, S. , & Dufour, A.‐B. (2007). The ade4 package: Implementing the duality diagram for ecologists. Journal of Statistical Software, 22(4), 1–20. 10.18637/jss.v022.i04

[ece311662-bib-0026] Dray, S. , Legendre, P. , & Peres‐Neto, P. R. (2006). Spatial modeling: A comprehensive framework for principal coordinate analysis of neighbour matrices (PCNM). Ecological Modelling, 196(3–4), 483–493. 10.1016/j.ecolmodel.2006.02.015

[ece311662-bib-0027] Duc, C. , Benoit, M. , Détourné, G. , Simon, L. , Poulet, A. , Jung, M. , Veluchamy, A. , Latrasse, D. , Le Goff, S. , Cotterell, S. , Tatout, C. , Benhamed, M. , & Probst, A. V. (2017). Arabidopsis ATRX Modulates H3.3 Occupancy and Fine‐Tunes Gene Expression. The Plant Cell, 29(7), 1773–1793. 10.1105/tpc.16.00877 28684426 PMC5559740

[ece311662-bib-0028] Earl, D. A. , & von Holdt, B. M. (2012). STRUCTURE HARVESTER: A website and program for visualizing STRUCTURE output and implementing the Evanno method. Conservation Genetics Resources, 4, 359–361. 10.1007/s12686-011-9548-7

[ece311662-bib-0029] Elshire, R. J. , Glaubitz, J. C. , Sun, Q. , Poland, J. A. , Kawamoto, K. , Buckler, E. S. , & Mitchell, S. E. (2011). A robust, simple genotyping‐by‐sequencing (GBS) approach for high diversity species. PLoS One, 6(5), e19379. 10.1371/journal.pone.0019379 21573248 PMC3087801

[ece311662-bib-0030] Engels, J. M. , & Thormann, I. (2020). Main challenges and actions needed to improve conservation and sustainable use of our crop wild relatives. Plants, 9(8), 968. 10.3390/plants9080968 32751715 PMC7463933

[ece311662-bib-0031] Fick, S. E. , & Hijmans, R. J. (2017). WorldClim 2: New 1‐km spatial resolution climate surfaces for global land areas. International Journal of Climatology, 37, 4302–4315. 10.1002/joc.5086

[ece311662-bib-0032] Forester, B. R. , Lasky, J. R. , Wagner, H. H. , & Urban, D. L. (2018). Comparing methods for detecting multilocus adaptation with multivariate genotype‐environment associations. Molecular Ecology, 27(9), 2215–2233. 29633402 10.1111/mec.14584

[ece311662-bib-0033] Frichot, E. , Mathieu, F. , Trouillon, T. , Bouchard, G. , & Francois, O. (2014). Fast and efficient estimation of individual ancestry coefficients. Genetics, 194(4), 973–983. 10.1534/genetics.113.160572 PMC3982712 24496008

[ece311662-bib-0034] Fujiki, Y. , Ito, M. , Nishida, I. , & Watanabe, A. (2001). Leucine and its keto acid enhance the coordinated expression of genes for branched‐chain amino acid catabolism in *Arabidopsis* under sugar starvation. FEBS Letters, 499(1–2), 161–165. 10.1016/S0014-5793(01)02536-4 11418132

[ece311662-bib-0035] Gaudet, P. , Livstone, M. S. , Lewis, S. E. , & Thomas, P. D. (2011). Phylogenetic‐based propagation of functional annotations within the gene ontology consortium. Briefings in Bioinformatics, 12(5), 449–462. 10.1093/bib/bbr042 21873635 PMC3178059

[ece311662-bib-0036] Gibson, M. J. S. , & Moyle, L. C. (2020). Regional differences in the abiotic environment contribute to genomic divergence within a wild tomato species. Molecular Ecology, 29(12), 2204–2217. 10.1111/mec.15477 32419208

[ece311662-bib-0037] Goudet, J. , & Jombart, T. (2022). hierfstat: Estimation and tests of hierarchical F‐Statistics . R Package Version 0.5‐11, https://CRAN.R‐project.org/package=hierfstat

[ece311662-bib-0038] Gramazio, P. , Alonso, D. , Arrones, A. , Villanueva, G. , Plazas, M. , Toppino, L. , Barchi, L. , Portis, E. , Ferrante, P. , Lanteri, S. , Rotino, G. L. , Giuliano, G. , Vilanova, S. , & Prohens, J. (2023). Conventional and new genetic resources for an eggplant breeding revolution. Journal of Experimental Botany, 74(20), 6285–6305. 10.1093/jxb/erad260 37419672

[ece311662-bib-0039] Gramazio, P. , Prohens, J. , Plazas, M. , Mangino, G. , Herraiz, F. J. , Garcia‐Fortea, E. , & Vilanova, S. (2018). Genomic tools for the enhancement of vegetable crops: A case in eggplant. Notulae Botanicae Horti Agrobotanici Cluj‐Napoca, 46(1), 1–13. 10.15835/nbha46110936

[ece311662-bib-0040] Gramazio, P. , Prohens, J. , Plazas, M. , Mangino, G. , Herraiz, F. J. , & Vilanova, S. (2017). Development and genetic characterization of advanced backcross materials and an introgression line population of *Solanum incanum* in a *S. melongena* background. Frontiers in Plant Science, 8, 1477. 10.3389/fpls.2017.01477 28912788 PMC5582342

[ece311662-bib-0041] Gramazio, P. , Yan, H. , Hasing, T. , Vilanova, S. , Prohens, J. , & Bombarely, A. (2019). Whole‐genome resequencing of seven eggplant (*Solanum melongena*) and one wild relative (*S. incanum*) accessions provides new insights and breeding tools for eggplant enhancement. Frontiers in Plant Science, 10, 1220. 10.3389/fpls.2019.01220 31649694 PMC6791922

[ece311662-bib-0042] Han, G. , Qiao, Z. , Li, Y. , Wang, C. , & Wang, B. (2021). The roles of CCCH zinc‐finger proteins in plant abiotic stress tolerance. International Journal of Molecular Sciences, 22(15), 8327. 10.3390/ijms22158327 34361093 PMC8347928

[ece311662-bib-0043] Haupt, M. , & Schmid, K. (2022). Using landscape genomics to infer genomic regions involved in environmental adaptation of soybean genebank accessions. *bioRxiv*. 10.1101/2022.02.18.480989 PMC12400605 40890586

[ece311662-bib-0044] Hengl, T. , Mendes de Jesus, J. , Heuvelink, G. B. M. , Ruiperez, G. M. , Kilibarda, M. , Blagotić, A. , Shangguan, W. , Wright, M. N. , Geng, X. , Bauer‐Marschallinger, B. , Guevara, M. A. , Vargas, R. , MacMillan, R. A. , Batjes, N. H. , Leenaars, J. G. B. , Ribeiro, E. , Wheeler, I. , Mantel, S. , & Kempen, B. (2017). SoilGrids250m: Global gridded soil information based on machine learning. PLoS One, 12(2), e0169748. 10.1371/journal.pone.0169748 28207752 PMC5313206

[ece311662-bib-0045] Hijmans, R. (2023). raster: Geographic data analysis and modeling . R Package Version 3.6‐14. https://CRAN.R‐project.org/package=raster

[ece311662-bib-0046] Hoban, S. , Kelley, J. L. , Lotterhos, K. E. , Antolin, M. F. , Bradburd, G. , Lowry, D. B. , Poss, M. L. , Reed, L. K. , Storfer, A. , & Whitlock, M. C. (2016). Finding the genomic basis of local adaptation: Pitfalls, practical solutions, and future directions. The American Naturalist, 188(4), 379–397. 10.1086/688018 PMC5457800 27622873

[ece311662-bib-0047] Huang, K. , Jahani, M. , Gouzy, J. , Legendre, A. , Carrere, S. , Miguel, J. , González Segovia, E. G. , Todesco, M. , Mayjonade, B. , Rodde, N. , Cauet, S. , Dufau, I. , Staton, S. E. , Pouilly, N. , Boniface, M. , Tapy, C. , Mangin, B. , Duhnen, A. , Gautier, V. , & Rieseberg, L. H. (2023). The genomics of linkage drag in inbred lines of sunflower. Proceedings of the National Academy of Sciences of the United States of America, 120(14), e2205783119. 10.1073/pnas.2205783119 36972449 PMC10083583

[ece311662-bib-0048] Ikezawa, N. , Iwasa, K. , & Sato, F. (2008). Molecular cloning and characterization of CYP80G2, a cytochrome P450 that catalyzes an intramolecular C‐C phenol coupling of (S)‐reticuline in magnoflorine biosynthesis, from cultured *Coptis japonica* cells. The Journal of Biological Chemistry, 283(14), 8810–8821. 10.1074/jbc.M705082200 18230623

[ece311662-bib-0049] Inouye, D. W. (2008). Effects of climate change on phenology, frost damage, and floral abundance of montane wildflowers. Ecology, 89(2), 353–362. 10.1890/06-2128.1 18409425

[ece311662-bib-0050] James, G. , Witten, D. , Hastie, T. , & Tibshirani, R. (2013). An introduction to statistical learning: With applications in R (1st ed.). Springer.

[ece311662-bib-0051] Joswig, J. S. , Wirth, C. , Schuman, M. C. , Kattge, J. , Reu, B. , Wright, I. J. , Sippel, S. D. , Rüger, N. , Richter, R. , Schaepman, M. E. , van Bodegom, P. M. , Cornelissen, J. H. C. , Díaz, S. , Hattingh, W. N. , Kramer, K. , Lens, F. , Niinemets, Ü. , Reich, P. B. , Reichstein, M. , … Mahecha, M. D. (2022). Climatic and soil factors explain the two‐dimensional spectrum of global plant trait variation. Nature Ecology & Evolution, 6, 36–50. 10.1038/s41559-021-01616-8 34949824 PMC8752441

[ece311662-bib-0052] Kacprzak, S. M. , Dahlqvist, A. , & Aken, O. V. (2020). The transcription factor ANAC017 is a key regulator of mitochondrial proteotoxic stress responses in plants. Philosophical Transactions of the Royal Society, B: Biological Sciences, 375(1801), 20190411. 10.1098/rstb.2019.0411 PMC7209956 32362262

[ece311662-bib-0053] Kamvar, Z. N. , Brooks, J. C. , & Grünwald, N. J. (2015). Novel R tools for analysis of genome‐wide population genetic data with emphasis on clonality. Frontiers in Genetics, 6, 208. 10.3389/fgene.2015.00208 26113860 PMC4462096

[ece311662-bib-0054] Kapazoglou, A. , Gerakari, M. , Lazaridi, E. , Kleftogianni, K. , Sarri, E. , Tani, E. , & Bebeli, P. J. (2023). Crop wild relatives: A valuable source of tolerance to various abiotic stresses. Plants, 12, 328. 10.3390/plants12020328 36679041 PMC9861506

[ece311662-bib-0055] Knapp, S. , Vorontsova, M. S. , & Prohens, J. (2013). Wild relatives of the eggplant (*Solanum melongena* L.: Solanaceae): New understanding of species names in a complex group. PLoS One, 8(2), e57039. 10.1371/journal.pone.0057039 23451138 PMC3579775

[ece311662-bib-0056] Kopelman, N. M. , Mayzel, J. , Jakobsson, M. , Rosenberg, N. A. , & Mayrose, I. (2015). Clumpak: A program for identifying clustering modes and packaging population structure inferences across K. Molecular Ecology Resources, 15(5), 1179–1191. 10.1111/1755-0998.12387 25684545 PMC4534335

[ece311662-bib-0057] Kurth, E. G. , Peremyslov, V. V. , Turner, H. L. , Makarova, K. S. , Iranzo, J. , Mekhedov, S. L. , Koonin, E. V. , & Dolja, V. V. (2017). Myosin‐driven transport network in plants. Proceedings of the National Academy of Sciences of the United States of America, 114(8), E1385–E1394. 10.1073/pnas.1620577114 28096376 PMC5338391

[ece311662-bib-0058] Lasky, J. R. , Des Mmarais, D. L. , McKay, J. K. , Richards, J. H. , Juenger, T. E. , & Keitt, T. H. (2012). Characterizing genomic variation of *Arabidopsis thaliana*: The roles of geography and climate. Molecular Ecology, 21, 5512–5529. 10.1111/j.1365-294X.2012.05709.x 22857709

[ece311662-bib-0059] Lasky, J. R. , Upadhyaya, H. D. , Ramu, P. , Deshpande, S. , Hash, C. T. , Bonnette, J. , Juenger, T. E. , Hyma, K. , Acharya, C. , Mitchell, S. E. , Buckler, E. S. , Brenton, Z. , Kresovich, S. , & Morris, G. P. (2015). Genome‐environment associations in sorghum landraces predict adaptive traits. Science Advances, 1(6), e1400218. 10.1126/sciadv.1400218 26601206 PMC4646766

[ece311662-bib-0060] Lee, J. , & Amasino, R. M. (2013). Two FLX family members are non‐redundantly required to establish the vernalization requirement in *Arabidopsis* . Nature Communications, 4(1), 1–9. 10.1038/ncomms3186 PMC3753012 23864009

[ece311662-bib-0061] Lee, S. , Jeong, H. J. , Kim, S. A. , Lee, J. , Guerinot, M. L. , & An, G. (2010). OsZIP5 is a plasma membrane zinc transporter in rice. Plant Molecular Biology, 73, 507–517. 10.1007/s11103-010-9637-0 20419467

[ece311662-bib-0062] Legendre, P. , & Legendre, L. (2012). Chapter 11 – Canonical analysis. In P. Legendre & L. Legendre (Eds.), Developments in environmental modelling. Numerical ecology (pp. 625–710). Elsevier.

[ece311662-bib-0063] Lei, L. , Poets, A. M. , Liu, C. , Wyant, S. R. , Hoffman, P. J. , Carter, C. K. , Shaw, B. G. , Li, X. , Muehlbauer, G. J. , Katagiri, F. , & Morrell, P. L. (2019). Environmental association identifies candidates for tolerance to low temperature and drought. G3: Genes, Genomes, Genetics, 9(10), 3423–3438. 10.1534/g3.119.400401 31439717 PMC6778781

[ece311662-bib-0064] Li, H. , & Durbin, R. (2009). Fast and accurate short read alignment with burrows‐wheeler transform. Bioinformatics, 25(14), 1754–1760. 10.1093/bioinformatics/btp324 19451168 PMC2705234

[ece311662-bib-0065] Li, H. , Handsaker, B. , Wysoker, A. , Fennell, T. , Ruan, J. , Homer, N. , Marth, G. , Abecasis, G. , Durbin, R. , & 1000 Genome Project Data Processing Subgroup . (2009). The sequence alignment/map format and SAMtools. Bioinformatics, 25(16), 2078–2079. 10.1093/bioinformatics/btp352 19505943 PMC2723002

[ece311662-bib-0066] Li, Y. , Chen, F. , Yang, Y. , Han, Y. , Ren, Z. , Li, X. , Soppe, W. J. J. , Cao, H. , & Liu, Y. (2023). The *Arabidopsis* pre‐mRNA 3' end processing related protein FIP1 promotes seed dormancy via the DOG1 and ABA pathways. The Plant Journal, 115, 494–509. 10.1111/tpj.16239 37035898

[ece311662-bib-0067] Li, Y. , Huang, Y. , Bergelson, J. , Nordborg, M. , & Borevitz, J. O. (2010). Association mapping of local climate‐sensitive quantitative trait loci in *Arabidopsis thaliana* . Proceedings of the National Academy of Sciences of the United States of America, 107(49), 21199–21204. 10.1073/pnas.1007431107 21078970 PMC3000268

[ece311662-bib-0068] Lin, P. , Wu, H. , Chan, K. , & Schafleitner, R. (2022). De novo SNP calling reveals the genetic differentiation and morphological divergence in genus *Amaranthus* . The Plant Genome, 15(2), e20206. 10.1002/tpg2.20206 35470587 PMC12806986

[ece311662-bib-0069] Luo, D. , Bernard, D. G. , Balk, J. , Hai, H. , & Cui, X. (2012). The DUF59 family gene AE7 acts in the cytosolic iron‐sulfur cluster assembly pathway to maintain nuclear genome integrity in *Arabidopsis* . The Plant Cell, 24(10), 4135–4148. 10.1105/tpc.112.102608 23104832 PMC3517241

[ece311662-bib-0070] Luu, K. , Bazin, E. , & Blum, M. G. (2016). pcadapt: An R package to perform genome scans for selection based on principal component analysis. Molecular Ecology Resources, 17(1), 67–77. 10.1111/1755-0998.12592 27601374

[ece311662-bib-0071] Malanson, G. P. , Zimmerman, D. L. , & Fagre, D. B. (2017). Distance and environmental difference in alpine plant communities. Physical Geography, 38(6), 489–505. 10.1080/02723646.2017.1327284

[ece311662-bib-0072] Manel, S. , Joost, S. , Epperson, B. K. , Holdereggere, R. , Storfer, A. , Rosenberg, M. S. , Scribner, K. T. , Bonin, A. , & Fortin, M. J. (2010). Perspectives on the use of landscape genetics to detect genetic adaptive variation in the field. Molecular Ecology, 19, 3760–3772. 10.1111/j.1365-294X.2010.04717.x 20723056

[ece311662-bib-0073] Mateo‐Bonmatí, E. , Esteve‐Bruna, D. , Juan‐Vicente, L. , Nadi, R. , Candela, H. , Lozano, F. M. , Ponse, M. R. , Pérez‐Pérez, J. M. , & Micol, J. L. (2005). INCURVATA11 and CUPULIFORMIS2 are redundant genes that encode epigenetic machinery components in *Arabidopsis* . The Plant Cell, 30(7), 1596–1616. 10.1105/tpc.18.00300 PMC6096603 29915151

[ece311662-bib-0074] Mdladla, K. , Dzomba, E. F. , & Muchadeyi, F. C. (2018). Landscape genomics and pathway analysis to understand genetic adaptation of south African indigenous goat populations. Heredity, 120(4), 369–378. 10.1038/s41437-017-0044-z 29422506 PMC5842216

[ece311662-bib-0075] Meng, X. , Li, L. , De Clercq, I. , Narsai, R. , Xu, Y. , Hartmann, A. , Claros, D. L. , Custovic, E. , Lewsey, M. G. , Whelan, J. , & Berkowitz, O. (2019). ANAC017 coordinates organellar functions and stress responses by reprogramming retrograde signaling. Plant Physiology, 180(1), 634–653. 10.1104/pp.18.01603 30872424 PMC6501098

[ece311662-bib-0076] Meyer, R. S. , Karol, K. G. , Little, D. P. , Nee, M. H. , & Litt, A. (2012). Phylogeographic relationships among Asian eggplants and new perspectives on eggplant domestication. Molecular Phylogenetics and Evolution, 63(3), 685–701. 10.1016/j.ympev.2012.02.006 22387533

[ece311662-bib-0077] Morente‐López, J. , García, C. , Draper, D. , & Iriondo, J. M. (2018). Geography and environment shape landscape genetics of Mediterranean alpine species *Silene ciliata* Poiret. (Caryophyllaceae). Frontiers in Plant Science, 9, 1698. 10.3389/fpls.2018.01698 30538712 PMC6277476

[ece311662-bib-0078] Müller, J. V. , Cockel, C. P. , Gianella, M. , & Guzzon, F. (2021). Treasuring crop wild relative diversity: Analysis of success from the seed collecting phase of the ‘Adapting agriculture to climate Change’ project. Genetic Resources and Crop Evolution, 68(7), 2749–2756. 10.1007/s10722-021-01229-x

[ece311662-bib-0079] Nadi, R. , Candela, H. , Lozano, F. M. , Ponce, M. R. , Manuel, J. , & Micol, J. L. (2018). INCURVATA11 and CUPULIFORMIS2 are redundant genes that encode epigenetic machinery components in *Arabidopsis* . The Plant Cell, 30(7), 1596–1616. 10.1105/tpc.18.00300 29915151 PMC6096603

[ece311662-bib-0080] Nieves‐Cordones, M. , Caballero, F. , Martínez, V. , & Rubio, F. (2012). Disruption of the *Arabidopsis thaliana* inward‐rectifier K^+^ channel AKT1 improves plant responses to water stress. Plant and Cell Physiology, 53(2), 423–432. 10.1093/pcp/pcr194 22210899

[ece311662-bib-0081] Oksanen, J. , Simpson, G. , Blanchet, F. , Kindt, R. , Legendre, P. , Minchin, P. , O'Hara, R. , Solymos, P. , Stevens, M. , Szoecs, E. , Wagner, H. , Barbour, M. , Bedward, M. , Bolker, B. , Borcard, D. , Carvalho, G. , Chirico, M. , De Caceres, M. , Durand, S. , … Weedon, J. (2022). vegan: Community ecology package . R package version 2.6‐4. https://CRAN.R‐project.org/package=vegan

[ece311662-bib-0082] Parniske, M. , Hammond‐Kosack, K. E. , Golstein, C. , Thomas, C. M. , Jones, D. A. , Harrison, K. , Wulff, B. B. H. , & Jones, J. D. G. (1997). Novel disease resistance specificities result from sequence exchange between tandemly repeated genes at the Cf‐4/9 locus of tomato. Cell, 91(6), 821–832. 10.1016/s0092-8674(00)80470-5 9413991

[ece311662-bib-0083] Pasbani, B. , Salimi, A. , Aliasgharzad, N. , & Hajiboland, R. (2020). Colonization with arbuscular mycorrhizal fungi mitigates cold stress through improvement of antioxidant defense and accumulation of protecting molecules in eggplants. Scientia Horticulturae, 272, 109575. 10.1016/j.scienta.2020.109575

[ece311662-bib-0084] Pebesma, E. (2018). Simple features for R: Standardized support for spatial vector data. The R Journal, 10(1), 439–446. 10.32614/RJ-2018-009

[ece311662-bib-0085] Peri, P. L. , Rosas, Y. M. , Ladd, B. , Toledo, S. , Lasagno, R. G. , & Martínez, P. G. (2019). Modeling soil nitrogen content in South Patagonia across a climate gradient, vegetation type, and grazing. Sustainability, 11(9), 2707. 10.3390/su11092707

[ece311662-bib-0086] Plazas, M. , González‐Orenga, S. , Nguyen, H. T. , Morar, I. M. , Fita, A. , Boscaiu, M. , Prohens, J. , & Vicente, O. (2022). Growth and antioxidant responses triggered by water stress in wild relatives of eggplant. Scientia Horticulturae, 293, 110685. 10.1016/j.scienta.2021.110685

[ece311662-bib-0087] Plazas, M. , Vilanova, S. , Gramazio, P. , Rodríguez‐Burruezo, A. , Fita, A. , Herraiz, F. J. , Ranil, R. , Fonseka, R. , Niran, L. , Fonseka, H. , Kouassi, B. , Kouassi, A. , Kouassi, A. , & Prohens, J. (2016). Interspecific hybridization between eggplant and wild relatives from different genepools. Journal of the American Society for Horticultural Science, 141, 34–44.

[ece311662-bib-0088] Pritchard, J. K. , Stephens, M. , & Donnelly, P. (2000). Inference of population structure using multilocus genotype data. Genetics, 155(2), 945–959. 10.1093/genetics/155.2.945 10835412 PMC1461096

[ece311662-bib-0089] Pyo, Y. J. , Gierth, M. , Schroeder, J. I. , & Cho, M. H. (2010). High‐affinity K+ transport in *Arabidopsis*: AtHAK5 and AKT1 are vital for seedling establishment and postgermination growth under low‐potassium conditions. Plant Physiology, 153(2), 863–875. 10.1104/pp.110.154369 20413648 PMC2879780

[ece311662-bib-0090] Qin, L. , Walk, T. C. , Han, P. , Chen, L. , Zhang, S. , Li, Y. , Hu, X. , Xie, L. , Yang, Y. , Liu, J. , Lu, X. , Yu, C. , Tian, J. , Shaff, J. E. , Kochian, L. V. , Liao, X. , & Liao, H. (2019). Adaption of roots to nitrogen deficiency revealed by 3D quantification and proteomic analysis. Plant Physiology, 179(1), 329–347. 10.1104/pp.18.00716 30455286 PMC6324228

[ece311662-bib-0091] Rakha, M. , Namisy, A. , Chen, R. , El‐Mahrouk, M. E. , Metwally, E. , Taha, N. , Prohens, J. , Plazas, M. , & Taher, D. (2020). Development of interspecific hybrids between a cultivated eggplant resistant to bacterial wilt (*Ralstonia solanacearum*) and eggplant wild relatives for the development of rootstocks. Plants, 9(10), 1405. 10.3390/plants9101405 33096943 PMC7589714

[ece311662-bib-0092] Rellstab, C. , Gugerli, F. , Eckert, A. J. , Hancock, A. M. , & Holderegger, R. (2015). A practical guide to environmental association analysis in landscape genomics. Molecular Ecology, 24, 4348–4370. 10.1111/mec.13322 26184487

[ece311662-bib-0093] Rellstab, C. , Zoller, S. , Walthert, L. , Lesur, I. , Pluess, A. R. , Graf, R. , Sperisen, C. , Kremer, A. , & Gugerli, F. (2016). Signatures of local adaptation in candidate genes of oaks (*Quercus* spp.) with respect to present and future climatic conditions. Molecular Ecology, 25(23), 5907–5924. 27759957 10.1111/mec.13889

[ece311662-bib-0094] Renzi, J. P. , Coyne, C. J. , Berger, J. , von Wettberg, E. , Nelson, M. , Ureta, S. , Hernández, F. , Smýkal, P. , & Brus, J. (2022). How could the use of crop wild relatives in breeding increase the adaptation of crops to marginal environments? Frontiers in Plant Science, 13, 886162. 10.3389/fpls.2022.886162 35783966 PMC9243378

[ece311662-bib-0095] Richardson, J. L. , Brady, S. P. , Wang, I. J. , & Spear, S. F. (2016). Navigating the pitfalls and promise of landscape genetics. Molecular Ecology, 25, 849–863. 10.1111/mec.13527 26756865

[ece311662-bib-0096] Ruckle, M. E. , Burgoon, L. D. , Lawrence, L. A. , Sinkler, C. A. , & Larkin, R. M. (2012). Plastids are major regulators of light signaling in *Arabidopsis* . Plant Physiology, 159(1), 366–390. 10.1104/pp.112.193599 22383539 PMC3375971

[ece311662-bib-0097] Samuel, M. A. , Mudgil, Y. , Salt, J. N. , Delmas, F. , Ramachandran, S. , Chilelli, A. , & Goring, D. R. (2008). Interactions between the S‐domain receptor kinases and AtPUB‐ARM E3 ubiquitin ligases suggest a conserved signaling pathway in *Arabidopsis* . Plant Physiology, 147(4), 2084–2095. 10.1104/pp.108.123380 18552232 PMC2492606

[ece311662-bib-0098] Sanjur, O. I. , Piperno, D. R. , Andres, T. C. , & Wessel‐Beaver, L. (2002). Phylogenetic relationships among domesticated and wild species of Cucurbita (Cucurbitaceae) inferred from a mitochondrial gene: Implications for crop plant evolution and areas of origin. Proceedings of the National Academy of Sciences of the United States of America, 99(1), 535–540. 11782554 10.1073/pnas.012577299 PMC117595

[ece311662-bib-0099] Shafiq, S. , Berr, A. , & Shen, W.‐H. (2014). Combinatorial functions of diverse histone methylations in *Arabidopsis thaliana* flowering time regulation. New Phytologist, 201, 312–322. 10.1111/nph.12493 24102415

[ece311662-bib-0100] Steane, D. A. , Potts, B. M. , McLean, E. , Prober, S. M. , Stock, W. D. , Vaillancourt, R. E. , & Byrne, M. (2014). Genome‐wide scans detect adaptation to aridity in a widespread forest tree species. Molecular Ecology, 23, 2500–2513. 10.1111/mec.12751 24750317

[ece311662-bib-0101] Stetter, M. G. , Thornton, K. , & Ross‐Ibarra, J. (2018). Genetic architecture and selective sweeps after polygenic adaptation to distant trait optima. PLoS Genetics, 14(11), e1007794. 30452452 10.1371/journal.pgen.1007794 PMC6277123

[ece311662-bib-0102] Syfert, M. M. , Castañeda‐Álvarez, N. P. , Khoury, C. K. , Särkinen, T. , Sosa, C. C. , Achicanoy, H. A. , Bernau, V. , Prohens, J. , Daunay, C. , & Knapp, S. (2016). Crop wild relatives of the brinjal eggplant (*Solanum melongena*): Poorly represented in genebanks and many species at risk of extinction. American Journal of Botany, 103(4), 635–651. 10.3732/ajb.1500539 27026215

[ece311662-bib-0103] Terés, J. , Busoms, S. , Martín, L. P. , Luís‐Villarroya, A. , Flis, P. , Álvarez‐Fernández, A. , Tolrà, R. , Salt, D. E. , & Poschenrieder, C. (2019). Soil carbonate drives local adaptation in *Arabidopsis thaliana* . Plant, Cell & Environment, 42(8), 2384–2398. 10.1111/pce.13567 PMC6663613 31018012

[ece311662-bib-0104] The UniProt Consortium . (2023). UniProt: The universal protein knowledgebase in 2023. Nucleic Acids Research, 51(D1), D523–D531. 10.1093/nar/gkac1052 36408920 PMC9825514

[ece311662-bib-0105] Tiwari, L. D. , & Grover, A. (2019). Cpn60β4 protein regulates growth and developmental cycling and has bearing on flowering time in *Arabidopsis thaliana* plants. Plant Science, 286, 78–88. 10.1016/j.plantsci.2019.05.022 31300145

[ece311662-bib-0106] Tripodi, P. , Rabanus‐Wallace, M. T. , Barchi, L. , Kale, S. , Esposito, S. , Acquadro, A. , & Stein, N. (2021). Global range expansion history of pepper (*Capsicum* spp.) revealed by over 10,000 GenBank accessions. Proceedings of the National Academy of Sciences, 118(34), e2104315118. 10.1073/pnas.2104315118 PMC8403938 34400501

[ece311662-bib-0107] Wang, W. , Liu, B. , Xu, M. , Jamil, M. , & Wang, G. (2015). ABA‐induced CCCH tandem zinc finger protein OsC3H47 decreases ABA sensitivity and promotes drought tolerance in *Oryza sativa* . Biochemical and Biophysical Research Communications, 464(1), 33–37. 10.1016/j.bbrc.2015.05.087 26047696

[ece311662-bib-0108] Weese, T. L. , & Bohs, L. (2010). Eggplant origins: Out of Africa, into the orient. Taxon, 59, 49–56.

[ece311662-bib-0109] WFO . (2023). World Flora online . http://www.worldfloraonline.org

[ece311662-bib-0110] Wickham, H. (2016). ggplot2: Elegant graphics for data analysis. Springer‐Verlag.

[ece311662-bib-0111] Yadav, S. , Stow, A. J. , & Dudaniec, R. Y. (2021). Microgeographical adaptation corresponds to elevational distributions of congeneric montane grasshoppers. Molecular Ecology, 30(2), 481–498. 10.1111/mec.15739 33217095

[ece311662-bib-0112] Yu, H. , Zhang, Y. , Moss, B. L. , Bargmann, B. O. , Wang, R. , Prigge, M. , Nemhauser, J. L. , & Estelle, M. (2015). Untethering the TIR1 auxin receptor from the SCF complex increases its stability and inhibits auxin response. Nature Plants, 1(3), 1–8. 10.1038/nplants.2014.30 PMC4520256 26236497

[ece311662-bib-0113] Yuan, H. , Pawlowski, E. G. , Yang, Y. , Sun, T. , Thannhauser, T. W. , Mazourek, M. , Schnell, D. , & Li, L. (2021). *Arabidopsis* ORANGE protein regulates plastid pre‐protein import through interacting with tic proteins. Journal of Experimental Botany, 72(4), 1059–1072. 10.1093/jxb/eraa528 33165598

[ece311662-bib-0114] Zhang, S. , Liu, Y. , Du, M. , Shou, G. , Wang, Z. , & Xu, G. (2022). Nitrogen as a regulator for flowering time in plant. Plant and Soil, 480, 1–29. 10.1007/s11104-022-05608-w

[ece311662-bib-0115] Zhang, Y. , Zhang, B. , Yan, D. , Dong, W. , Yang, W. , Li, Q. , Zeng, L. , Wang, J. , Wang, L. , Hicks, L. M. , & He, Z. (2011). Two *Arabidopsis* cytochrome P450 monooxygenases, CYP714A1 and CYP714A2, function redundantly in plant development through gibberellin deactivation. The Plant Journal, 67(2), 342–353. 10.1111/j.1365-313X.2011.04596.x 21457373

[ece311662-bib-0116] Zhao, Z. , Yu, Y. , Meyer, D. , Wu, C. , & Shen, W. H. (2005). Prevention of early flowering by expression of FLOWERING LOCUS C requires methylation of histone H3 K36. Nature Cell Biology, 7(12), 1256–1260. 10.1038/ncb1329 16299497

[ece311662-bib-0117] Zsigmond, L. , Szarka, A. , Darula, Z. , Medzihradszky, K. F. , Koncz, C. , Koncz, Z. , & Szabados, L. (2008). *Arabidopsis* PPR40 connects abiotic stress responses to mitochondrial electron transport. Plant Physiology, 146(4), 1721–1737. 10.1104/pp.107.111260 18305213 PMC2287346

