# Supplementary material for: Landscape genomics reveals genetic signals of environmental adaptation of African wild eggplants
Source: Ecol Evol. 2024 Jul 9;14(7):e11662. doi: 10.1002/ece3.11662 (PMC11232056; doi:10.1002/ece3.11662)
Supplement: Supplementary file 1 — Figure S1. [file ECE3-14-e11662-s001.docx]

## Supporting Information

Article title:

**Landscape genomics reveals genetic signals of environmental adaptation of African wild eggplant**

Authors:

Emmanuel O. Omondi^1^, Chen-Yu Lin^2^, Shu-Mei Huang^2^, Cheng-An Liao^4^, Ya-Ping Lin^2^, Ricardo Oliva^3^, Maarten van Zonneveld^1^

^1^Genetic Resources and Seed Unit, World Vegetable Center, Headquarters, 60 Yi-Min Liao, Shanhua, Tainan 74151, Taiwan

^2^Biotechnology, World Vegetable Center, Headquarters, 60 Yi-Min Liao, Shanhua, Tainan 74151, Taiwan

^3^Plant Pathology, World Vegetable Center, Headquarters, 60 Yi-Min Liao, Shanhua, Tainan 74151, Taiwan

^4^Department of Horticulture, National Taiwan University, Taipei, Taiwan

**Correspondence email (**[**maarten.vanzonneveld@worldveg.org**](mailto:maarten.vanzonneveld@worldveg.org) **;** [**Emmanuel.omondi@worldveg.org**](mailto:Emmanuel.omondi@worldveg.org) **)**

The following Supporting Information is available for this article:

**
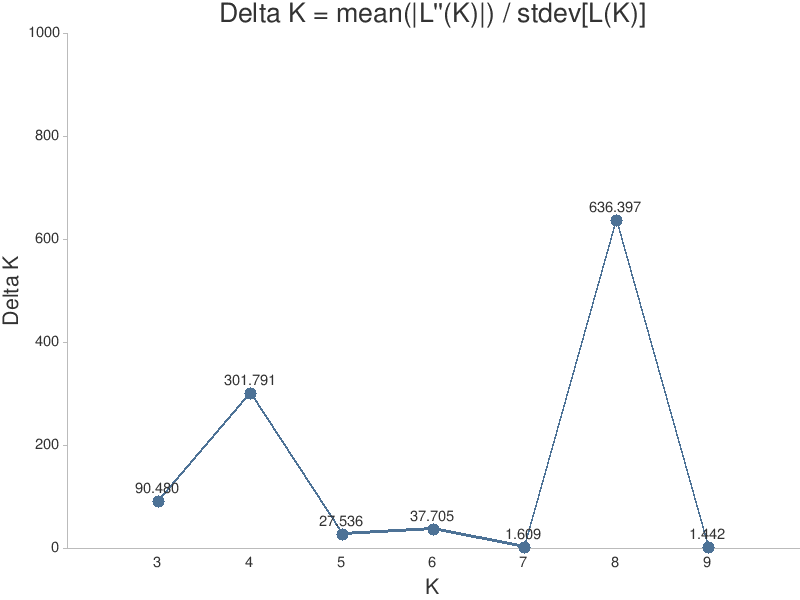
**

**Figure S1:** The highest delta K indicates the optimal K=8 obtained from the structure harvester results from the STRUCTURE analysis of the eggplant crop wild relative accessions.


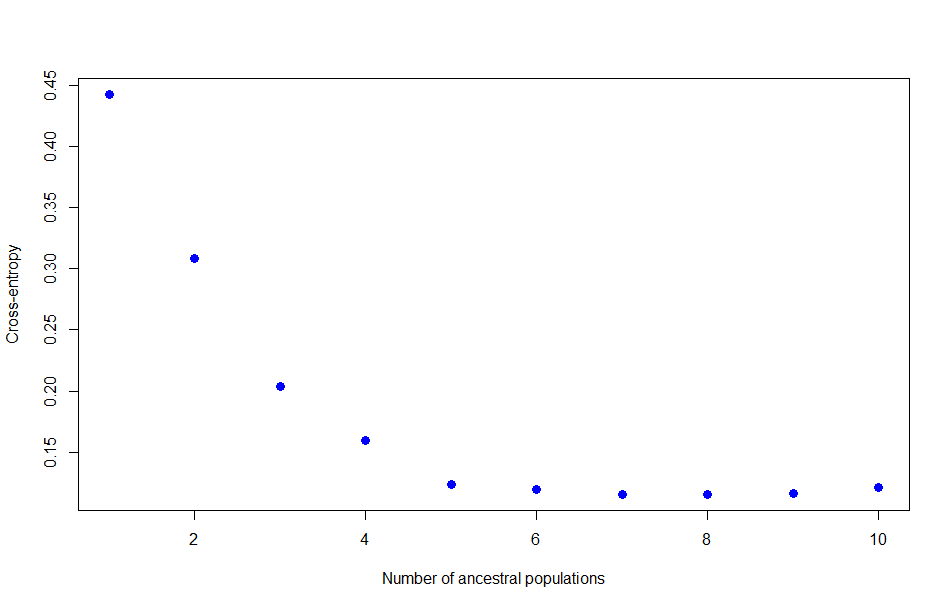


**Figure S2:** Cross-entropy plot of the snmf estimation of the ancestry coefficients. The estimated optimal K=5 (indicated with a red circle) indicates the curve's constant nature after this point.


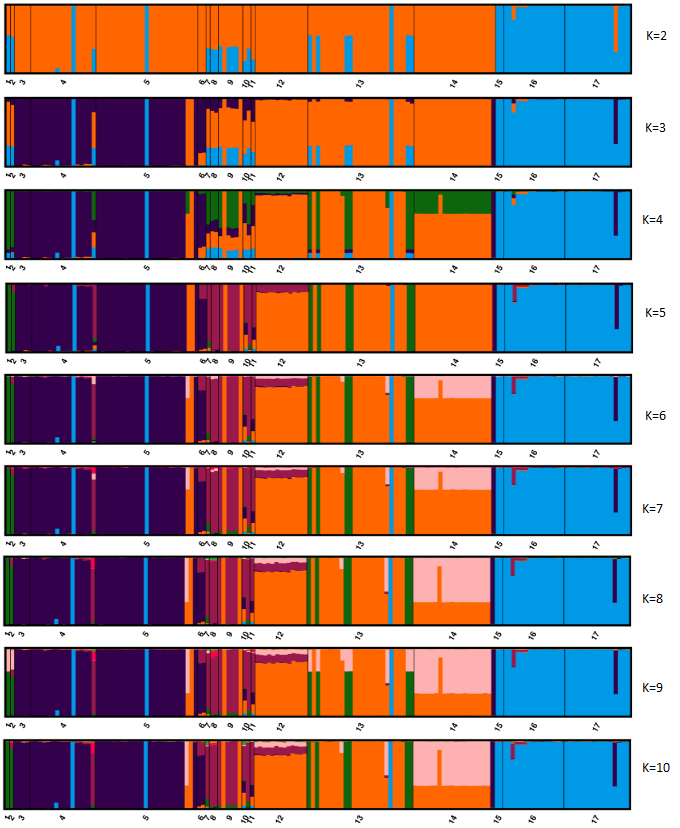


**Figure S3**: Inference of population structure bar plots estimated using STRUCTURE at K=2 to 10. The X-axis represents the individuals. The numbers represent the species assigned as populations in the STRUCTURE analysis. 1: *S. aculeastrum*; 2: *S. phoxocarpum*; 3: *S. eathiopicum*; 4: *S. anguivi*; 5: *S. anomalum*; 6: *S. mauense*: 7: *S. arundo*; 8: *S. aculeatissimum*; 9: *S. coagulans*; 10: *S. setaceum*; 11: *S. nigriviolaceum*; 12: *S. campylacanthum*; 13: *S. ceraciferum*; 14: *S. incanum*; 15: *S. dasyanthum*; 16: *S. dasyphyllum*; 17: *S. macrocarpon*.

**
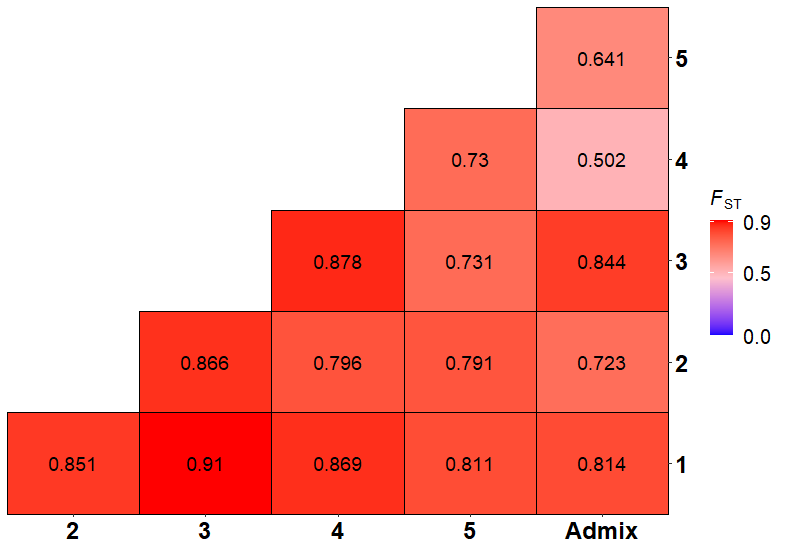
**

**Figure S4**: Pairwise *F_ST_* values between the groups based on the SNPs. The Numbers designate groups detected by the population structure analysis. The main species for every group included Groups 1 – *S. anomalum*, *S. incanum*, and *S. aethiopicum*; Group 2 - *S. macrocarpon*, *S. dasyphyllum*, *S. anomalum*, and *S. incanum*; Group 3 - *S. cerasiferum* and *S. anguivi*; Group 4 - *S. anguivi* and *S. anomalum*; and Group 5 – *S. campylacanthum*. Groups 1 represent the Coagulans, Acanthophora, and Arundo clades; Groups 2 and 5 represent the Anguivi and Giganteum clades, while Groups 3 and 4 represent the Melongena and Aculeastrum clades. Admixtures were observed mainly in groups 1 and 3 for accessions of *S. incanum*, *S. cerasiferum*, *S. coagulans*, and *S. nigriviolaceum* species.


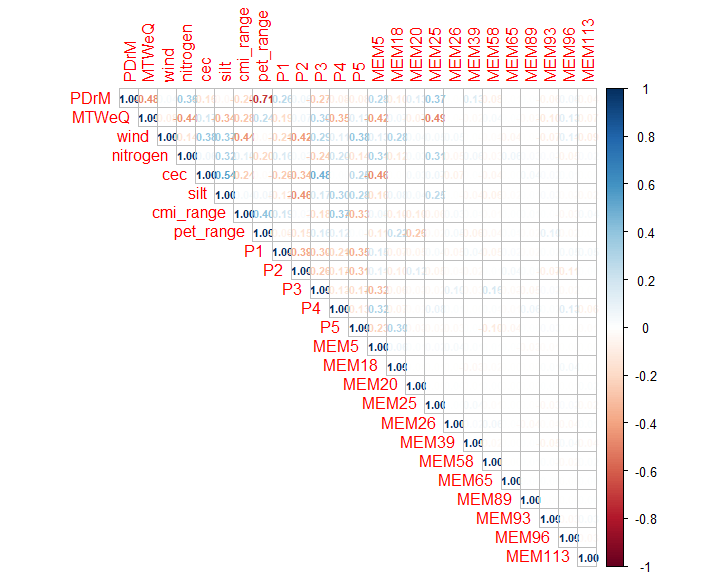


**Figure S5**: Correlation plot for all the variables. Environmental (climate and soil) - (PDrM - precipitation of the driest quarter, MTWeQ - mean temperature of the wettest quarter, wind - wind speed, cmi_range - annual range of monthly climate moisture index, pet_range - annual range of potential evapotranspiration, nitrogen - soil nitrogen content, silt - soil silt content, cec - cation exchange capacity), Ancestry coefficients (P1-P5) and Moran’s eigenvector maps (MEMs).


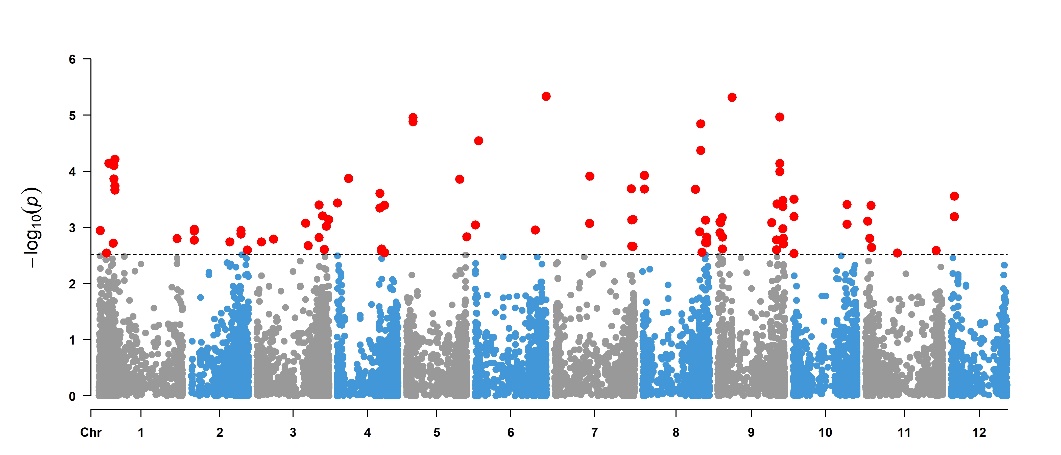

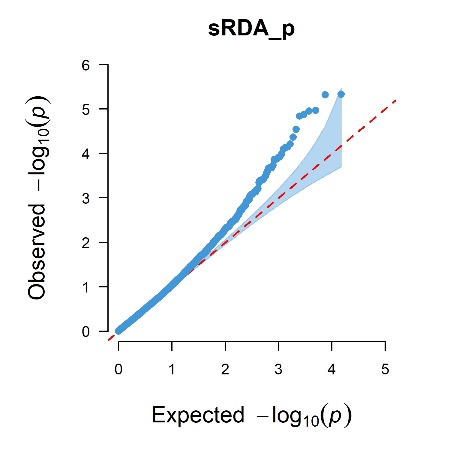


**A**


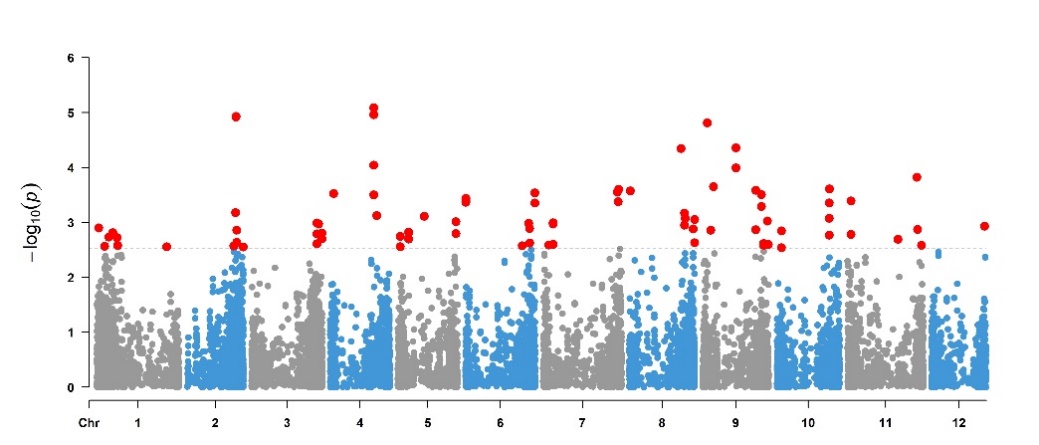


**B**


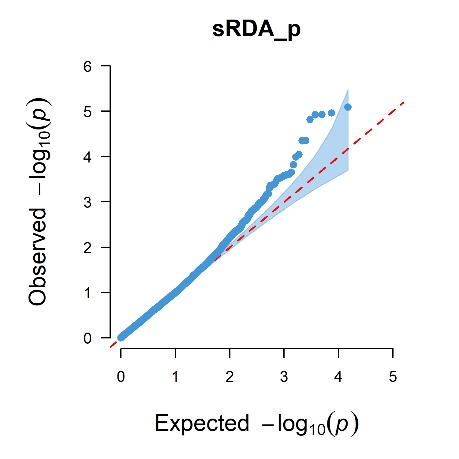

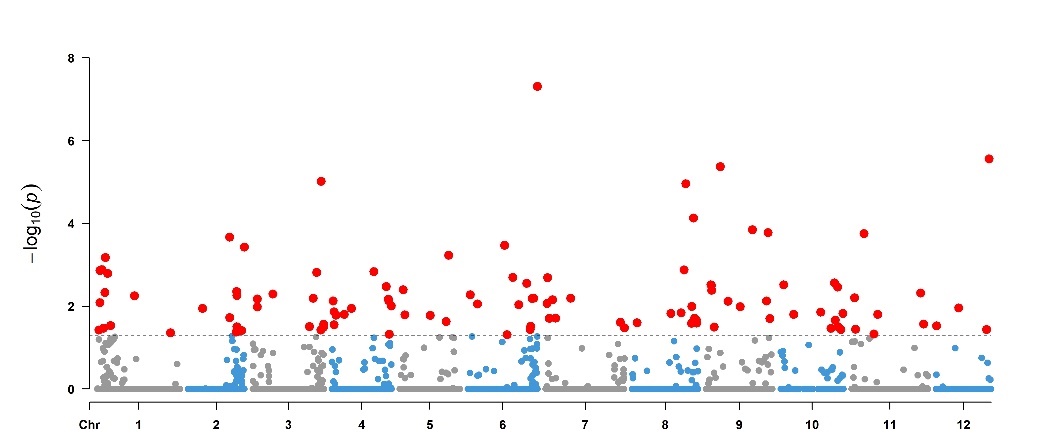

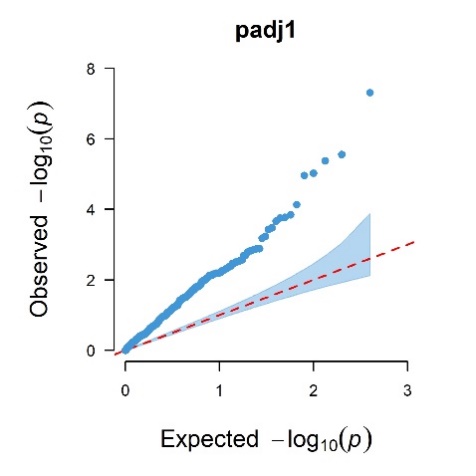


**C**

**Figure S6:** Manhattan plots correspond to the methods of outlier SNP detection: simple RDA on environmental data only (climate and soil)(A), partial RDA conditioned on population structure(B), and PCAdapt (C). Signiﬁcant SNPs are highlighted as red dots.


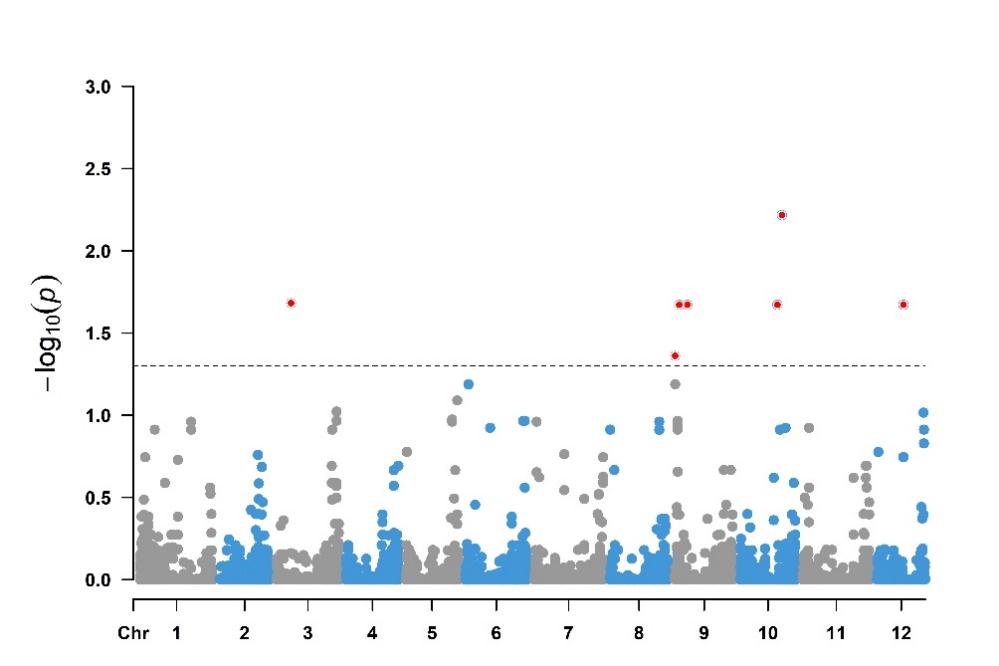

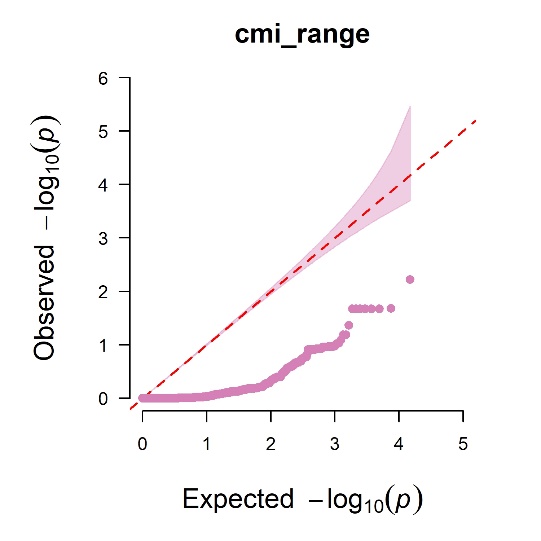

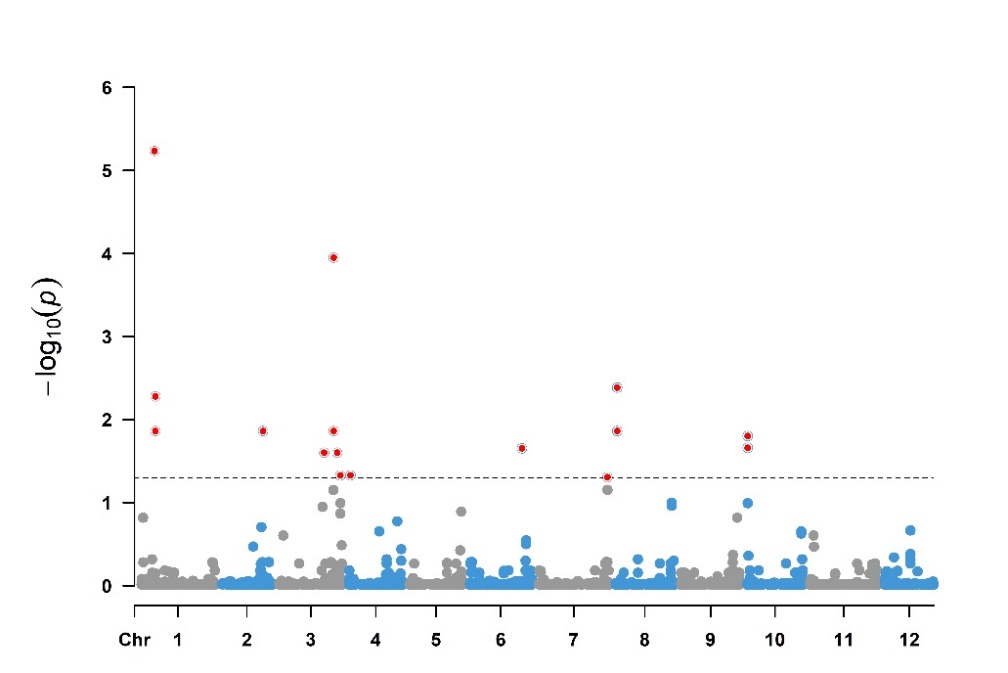

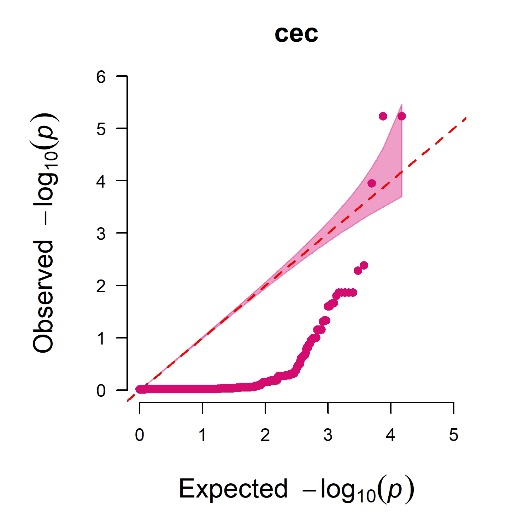

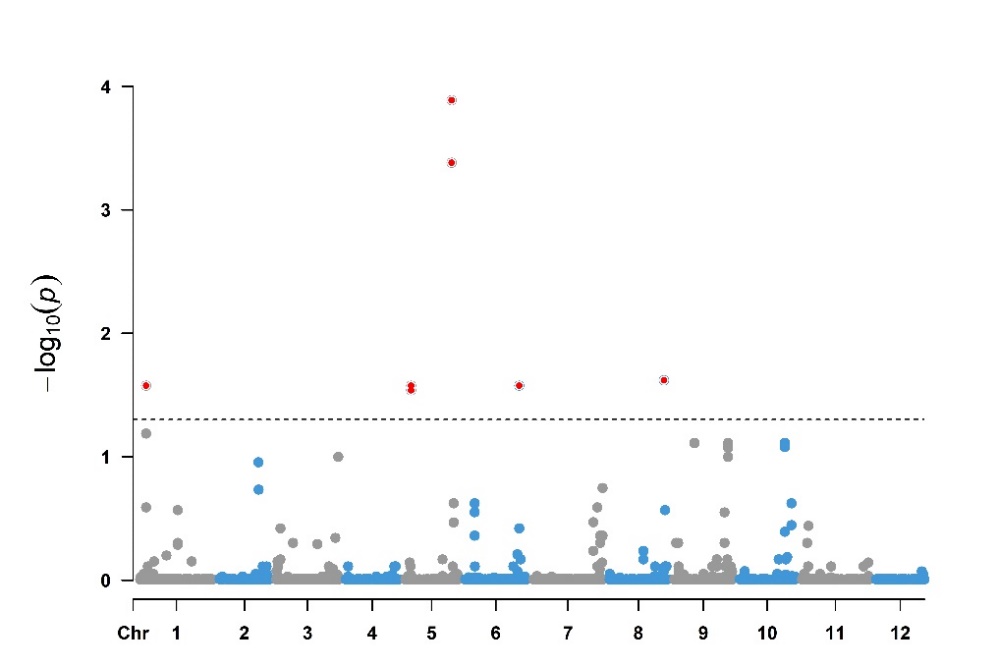

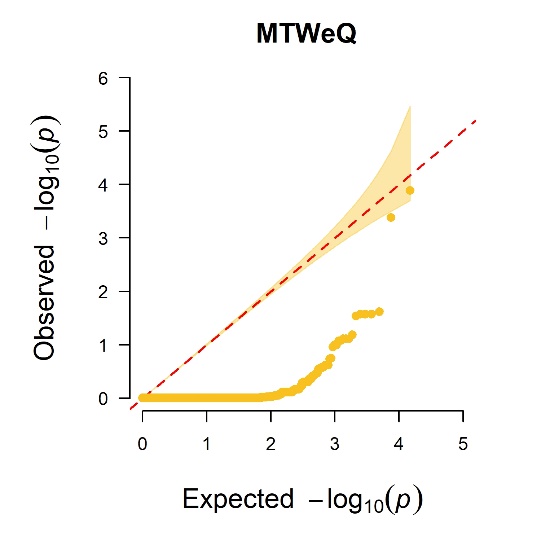

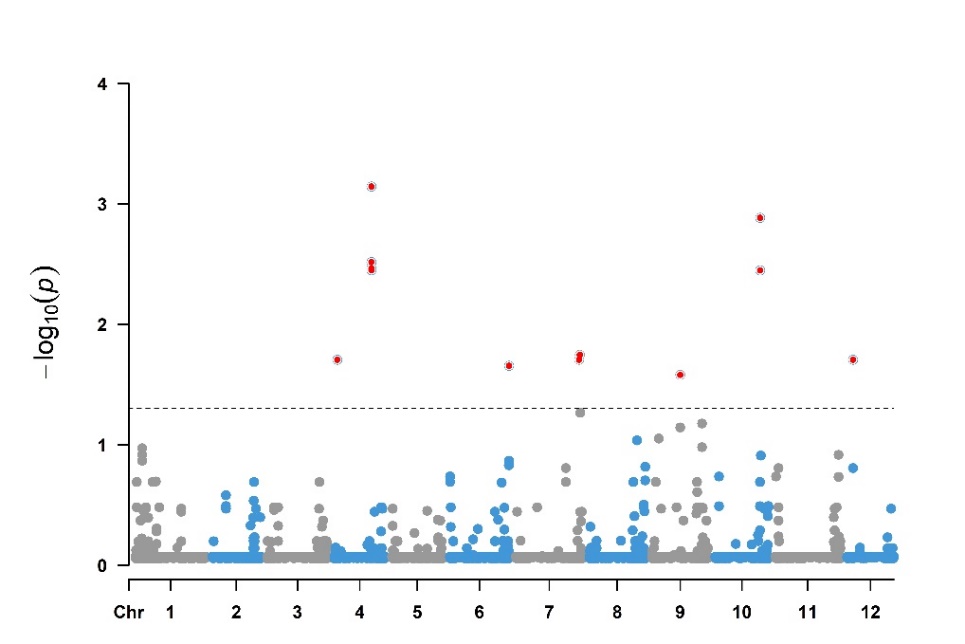

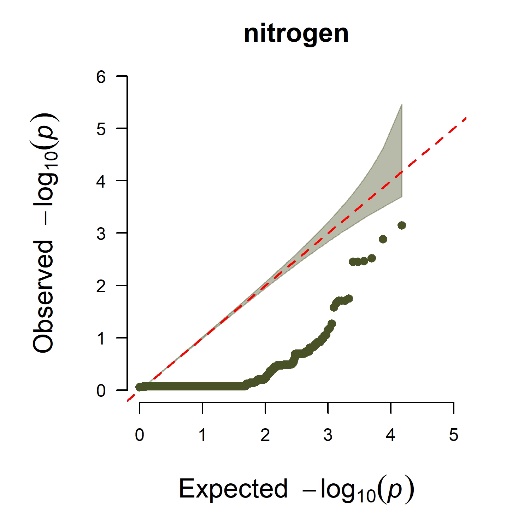

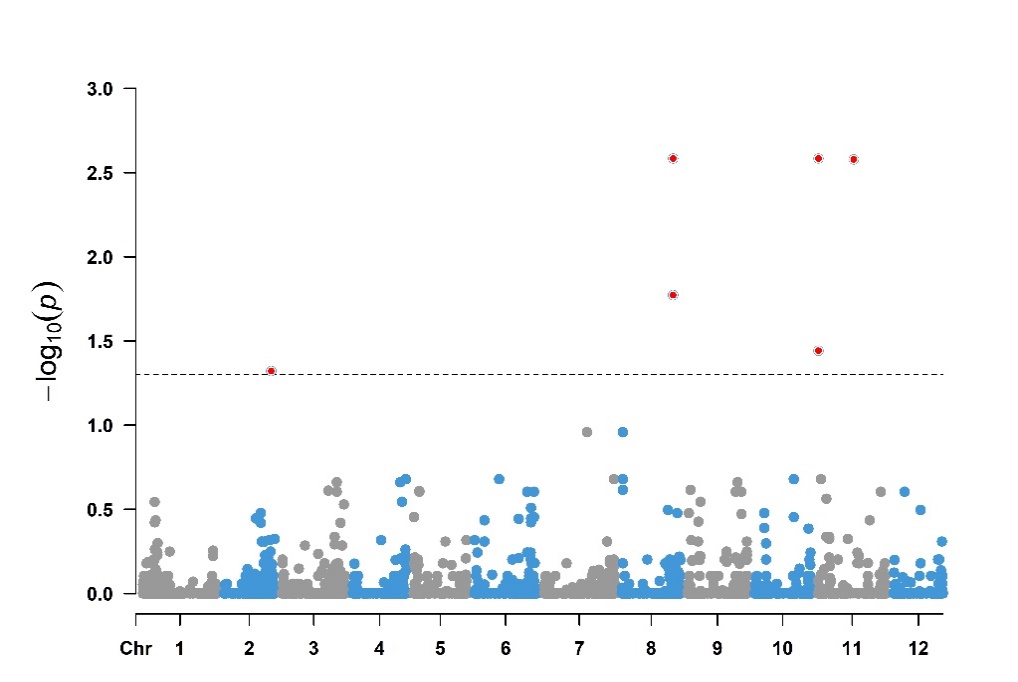

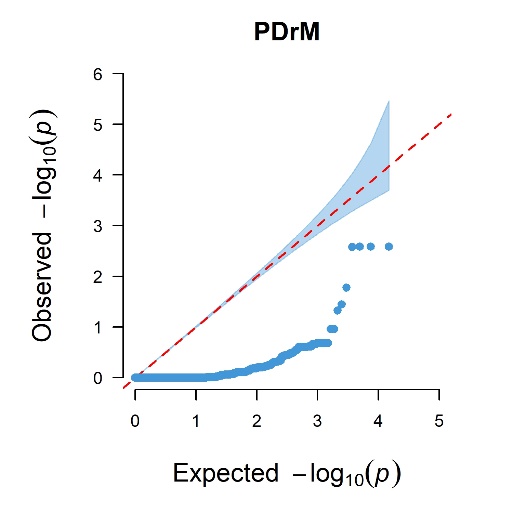

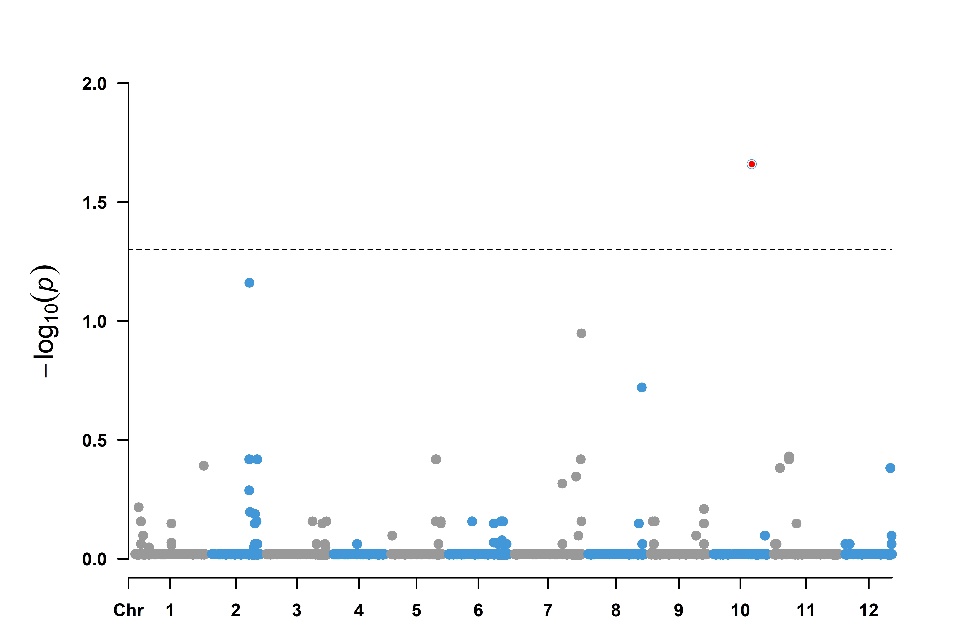

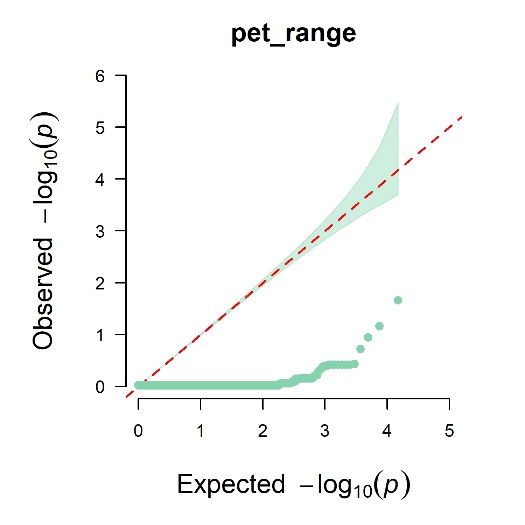

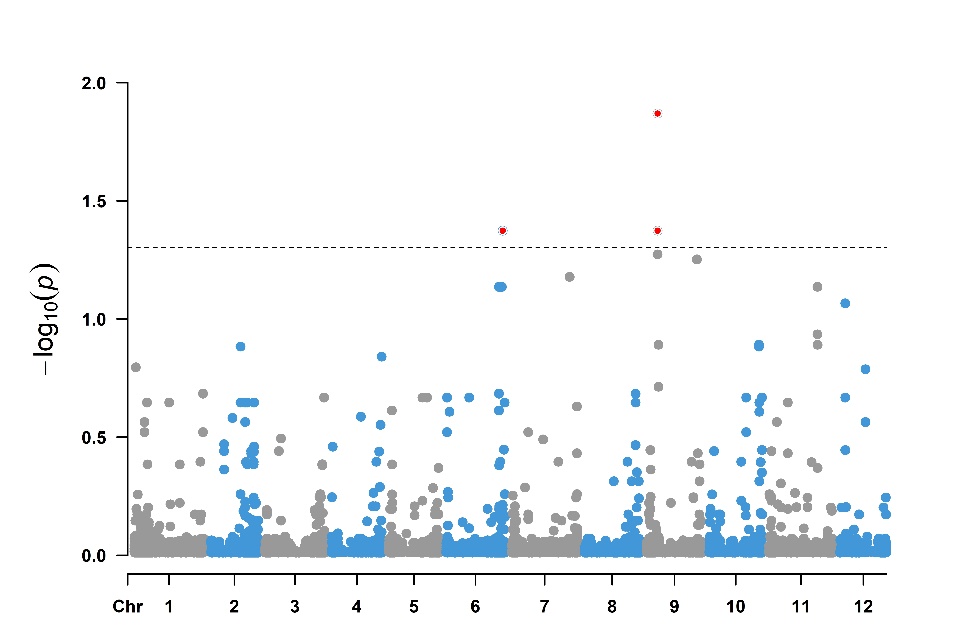

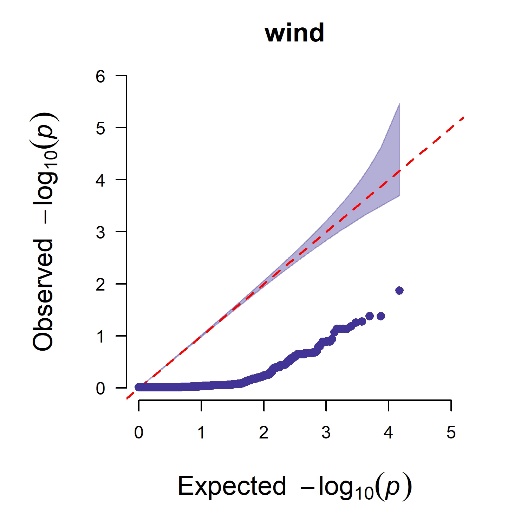

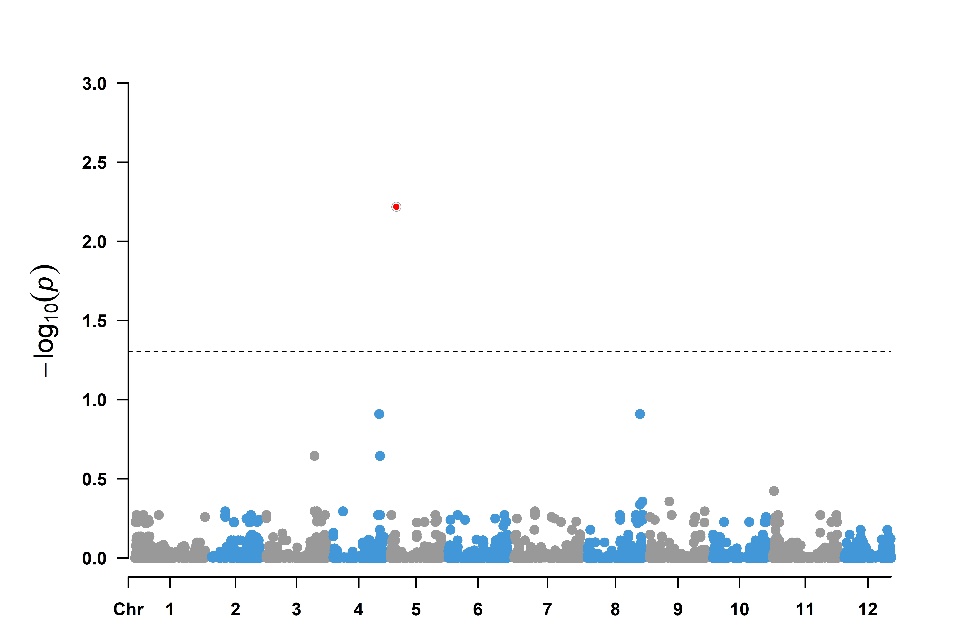

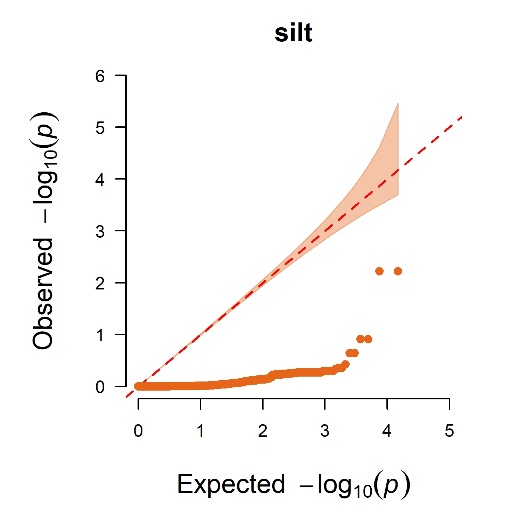


**Figure S7:** Manhattan plots correspond to the eight environmental variables (PDrM - precipitation of the driest quarter; MTWeQ - mean temperature of the wettest quarter; wind - wind speed; cmi_range - annual range of monthly climate moisture index; pet_range - annual range of potential evapotranspiration; nitrogen - soil nitrogen content; silt - soil silt content; cec - cation exchange capacity) correlated to the outlier SNP detected by the LFMM method. Signiﬁcant SNPs are highlighted as red dots.


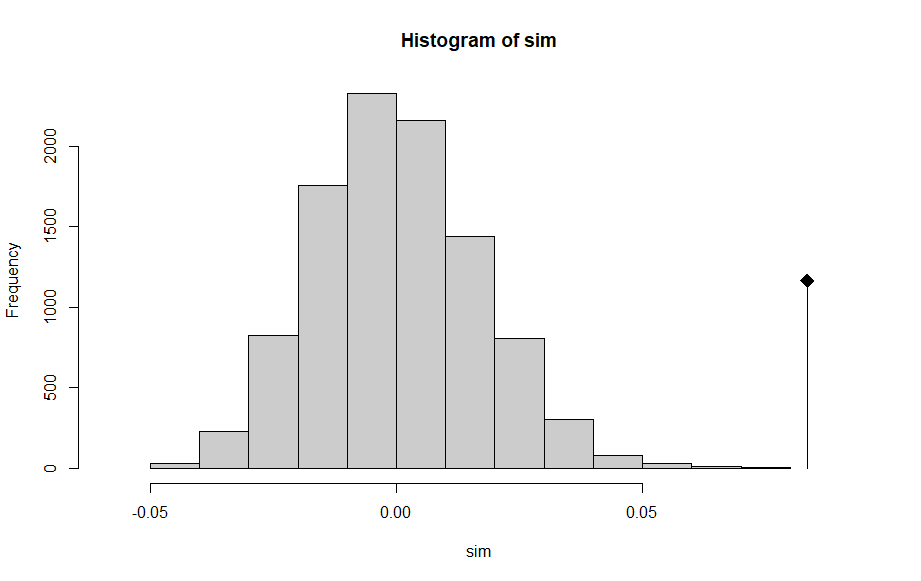


**Figure S8:** Results of Mantel test between spatial distances and environmental matrix. Spearman correlation coefficients (r) and associated p-values were calculated. Environmental distance is represented by Euclidean distance in terms of the eight environmental variables (PDrM - precipitation of the driest quarter; MTWeQ - mean temperature of the wettest quarter; wind - wind speed; cmi_range - annual range of monthly climate moisture index; pet_range - annual range of potential evapotranspiration; nitrogen - soil nitrogen content; silt - soil silt content; cec - cation exchange capacity).
